# A systematic review and meta-analysis of artificial intelligence versus clinicians for skin cancer diagnosis

**DOI:** 10.1038/s41746-024-01103-x

**Published:** 2024-05-14

**Authors:** Maria Paz Salinas, Javiera Sepúlveda, Leonel Hidalgo, Dominga Peirano, Macarena Morel, Pablo Uribe, Veronica Rotemberg, Juan Briones, Domingo Mery, Cristian Navarrete-Dechent

**Affiliations:** 1 Department of Dermatology, Escuela de Medicina, Pontificia Universidad Católica de Chile, Santiago, Chile; 2 Universidad Catolica-Evidence Center, Cochrane Chile Associated Center, Pontificia Universidad Católica de Chile, Santiago, Chile; 3 Melanoma and Skin Cancer Unit, Escuela de Medicina, Pontificia Universidad Católica de Chile, Santiago, Chile; 4 Dermatology Service, Department of Medicine, Memorial Sloan Kettering Cancer Center, New York, NY USA; 5 Department of Oncology, Escuela de Medicina, Pontificia Universidad Católica de Chile, Santiago, Chile; 6 Department of Computer Science, Pontificia Universidad Católica de Chile, Santiago, Chile

**Keywords:** Skin cancer, Translational research, Melanoma

## Abstract

Scientific research of artificial intelligence (AI) in dermatology has increased exponentially. The objective of this study was to perform a systematic review and meta-analysis to evaluate the performance of AI algorithms for skin cancer classification in comparison to clinicians with different levels of expertise. Based on PRISMA guidelines, 3 electronic databases (PubMed, Embase, and Cochrane Library) were screened for relevant articles up to August 2022. The quality of the studies was assessed using QUADAS-2. A meta-analysis of sensitivity and specificity was performed for the accuracy of AI and clinicians. Fifty-three studies were included in the systematic review, and 19 met the inclusion criteria for the meta-analysis. Considering all studies and all subgroups of clinicians, we found a sensitivity (Sn) and specificity (Sp) of 87.0% and 77.1% for AI algorithms, respectively, and a Sn of 79.78% and Sp of 73.6% for all clinicians (overall); differences were statistically significant for both Sn and Sp. The difference between AI performance (Sn 92.5%, Sp 66.5%) vs. generalists (Sn 64.6%, Sp 72.8%), was greater, when compared with expert clinicians. Performance between AI algorithms (Sn 86.3%, Sp 78.4%) vs expert dermatologists (Sn 84.2%, Sp 74.4%) was clinically comparable. Limitations of AI algorithms in clinical practice should be considered, and future studies should focus on real-world settings, and towards AI-assistance.

## Introduction

Skin cancer is the most common neoplasm worldwide. Early detection and diagnosis are critical for the survival of affected patients. For skin cancer detection in early stages, a complete physical examination is of paramount importance; however, visual inspection is often not sufficient, and less than one quarter of U.S. patients will have a dermatologic examination in their lifetime^1^. Dermoscopy is a diagnostic tool, which allows for improved recognition of numerous skin lesions when compared to naked eye examination alone; however, this improvement depends on the level of training and experience of clinicians^2^. In recent years, advances have been made in noninvasive tools to improve skin cancer diagnostic performance, including the use of artificial intelligence (AI) for clinical and/or dermoscopic image diagnosis in dermatology.

Convolutional neural networks (CNN) is a type of machine learning (ML) that simulates the processing of biological neurons and is the state-of-the-art network for pattern recognition in medical image analysis^1^^,2^. As diagnosis in dermatology relies heavily on both clinical and dermoscopic image recognition, the use of CNN has the potential to collaborate or improve diagnostic performance. Studies have been published demonstrating that CNN-based AI algorithms can perform similarly or even outperform specialists for skin cancer diagnosis^3^. This has created an ‘AI revolution’ in the field of skin cancer diagnosis. Recently, a few dermatology AI systems have been CE (*Conformité Européenne*) approved by the European Union and are use in practice making of paramount importance to understand the data behind these algorithms^4^.

While there have been relevant systematic reviews performed in the past few years, the importance of this work which combines a high-quality systematic review with a meta-analysis is that it quantitatively asks the question of where we are with AI for skin cancer detection. The main objective of this study was to perform a systematic review and meta-analysis to critically evaluate the evidence published to date on the performance of AI algorithms in skin cancer classification in comparison with clinicians.

## Methods

### Guidelines followed

**Fig. 1:**
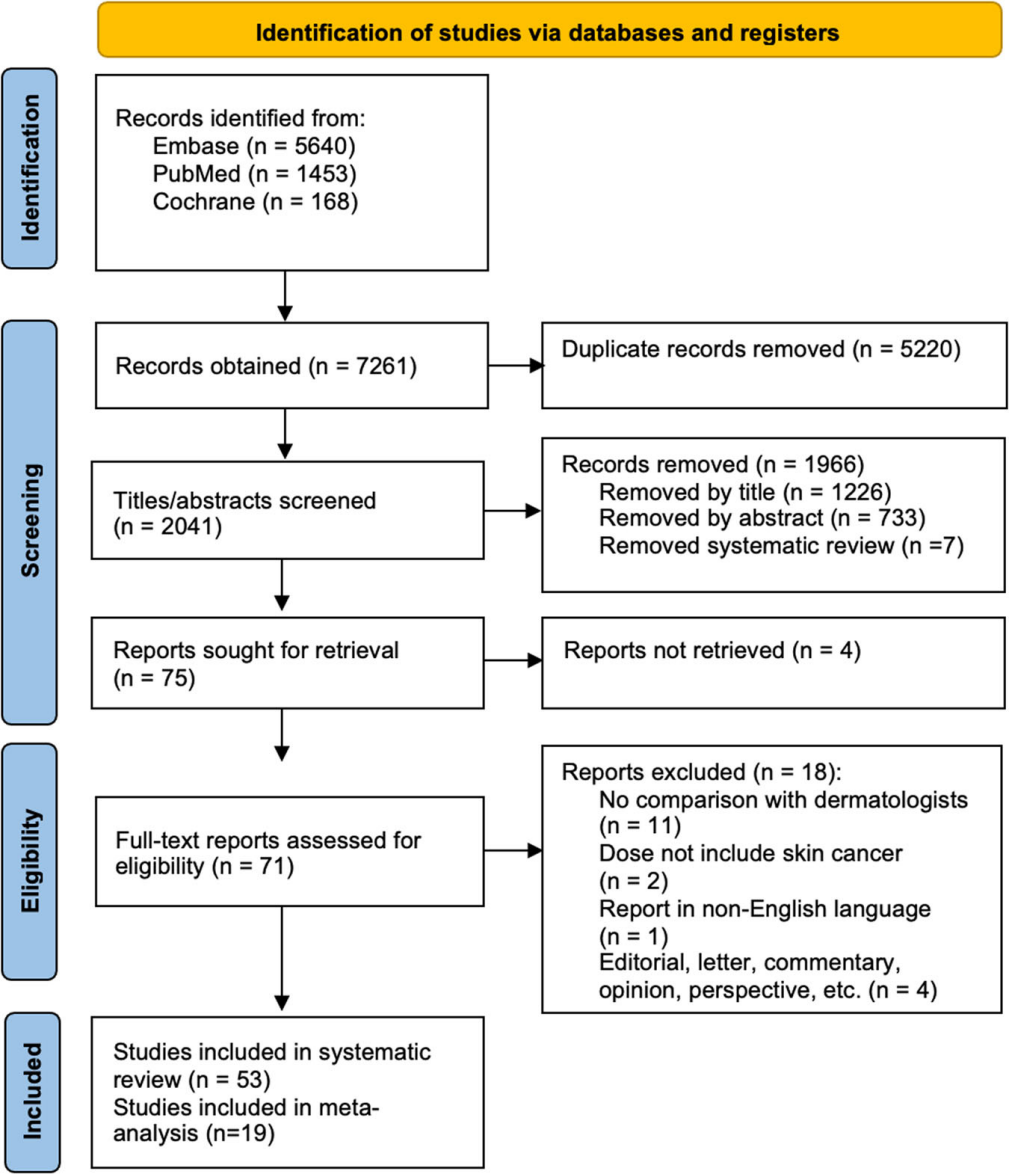
PRISMA flow diagram of included studies.

This systematic review was based on the PRISMA guidelines. A flow chart diagram is presented in Fig. 1. The present study has also been registered in the Prospective Register of Systematic Reviews (PROSPERO) System (PROSPERO ID: CRD42022368285).

### Search strategy

Three electronic databases, PubMed, Embase, and Cochrane library were searched by a librarian (J.M.). Studies published up to August 2022 were included. We uploaded all the titles and abstracts retrieved by electronic searching into Rayyan and removed any duplicate. Then we collected all the full texts of the studies that met the inclusion criteria based on the title or abstract for detailed inspection. Two reviewers (M.P.S. and J.S.) independently assessed the eligibility of the retrieved papers and resolved any discrepancies through discussion.

### Study population—selection

The following PICO (Population, Intervention or exposure, Comparison, Outcome) elements were applied as inclusion criteria for the systematic review: (i) Population: Images of patients with skin lesions, (ii) Intervention: Artificial intelligence diagnosis/classification, (iii) Comparator: Diagnosis/ classification by clinicians, (iv) Outcome: Diagnosis of skin lesions. Only primary studies comparing the performance of artificial intelligence versus dermatologists or clinicians were included.

Studies about diagnosis of inflammatory dermatoses, without extractable data, non-English publications, or animal studies, were excluded.

### Data extraction

For studies fulfilling the inclusion criteria, two independent reviewers extracted data in a standardized and predefined form. The following data were extracted and recorded: (i) Database (ii) Title, (iii) Year of publication, (iv) Author, (v) Journal, (vi) Prospective vs retrospective study, (vii) Image database used for training and internal vs external dataset for testing (viii) Type of images included: clinical and/or dermoscopy, (ix) Histopathology confirmation of diagnosis, (x) Inclusion of clinical information, (xi) Number and expertise of participants (experts dermatologists, non-expert dermatologists, and generalists), (xii) Name and type of AI algorithm, (xiii) Included diagnosis, (xiv) Statistics on diagnostic performance (sensitivity [Sn], specificity [Sp], receiver operating characteristic [ROC] curve, area under the curve [AUC]). The main comparisons conducted were diagnostic performance of the AI algorithm compared with clinician diagnostic performance. When available, the change in diagnostic performance of dermatologists with the support of the AI algorithm was included, as well as the change in diagnostic performance after including clinical data (data in supplementary material).

### Risk of bias assessment

Two review authors independently assessed the quality of the studies included and the risk of bias using QUADAS-2^5^. Based on the questions, we classified each QUADAS-2 domain as low (0), high (1) or unknown (2) risk of bias.

### Meta-analysis

Nineteen out of 53 studies were included in the meta-analysis. The studies met the following criteria: dermoscopic images only, diagnosis of skin cancer, dichotomous classification (benign/malignant, melanoma/nevus), extractable data from the original article (to calculate true positives [TP], false positives [FP], true negatives [TN], and false negatives [FN]), distinction in level of expertise of clinicians (experts dermatologists vs non-expert dermatologists vs generalists). For study purposes and to obtain a global estimate, we grouped all levels of clinical expertise as ‘overall clinicians’. During data processing, two extra analysis that were not pre-specified in the PROSPERO protocol were performed: clinician vs AI algorithms in prospective vs retrospective studies and internal vs external test (validation) sets, respectively. Internal vs external test sets were defined according to Cabitza^6^ and Shung et al.^7^. ‘Internal test set’ was defined as a non-overlapping, ‘held out’ subset of the original patient group data that was not used for AI algorithm development and training, used to test the AI model. ‘External test set’ was defined as a set of new data originating from different cohorts, facilities, or repositories other than the data used for model development and training (e.g., dataset originated in different country or institution). Two investigators classified included studies into internal vs external test sets. If both internal and external test sets were used, we classified them as external for study purposes. We decided to perform these non-pre-specified analysis given the relevance of the results for understanding of the data^8^.

We extracted binary diagnostic accuracy data and constructed contingency tables to calculate Sn and Sp. We conducted a meta-analysis of studies providing 2 ×2 tables to estimate the accuracy of AI and clinicians (confirmatory approach). If an included study provided various 2 ×2 tables, we assumed these data to be independent from each other. We performed a hierarchical summary receiver operating characteristic (HSROC) as well as a bivariate model of the accuracy of AI and clinicians. ROC curves were constructed to simplify the plotting of graphical summaries of fitted models. A likelihood ratio test was used to compare models. A p-value less than 0.05 was considered statistically significant. Analyses were performed using Stata 17.0 statistics software package (codes in supplementary material).

## Results

A total of 53 comparative studies (since Piccolo et al. in 2002^9^) fulfilled the inclusion criteria (Fig. 1). Most of the studies focused on dermoscopic images (*n* = 31), followed by clinical images (*n* = 14), or both (*n* = 8). Detailed extracted data is shown in Table 1 for dermoscopic imaging studies, Table 2 for clinical imaging studies, and Table 3 for clinical and dermoscopic imaging studies.

**Table 1:** Included studies general characteristics, dataset used, and performance evaluating dermoscopy

Author	Database	Training	Test set	I/E	Design	HP	CD	Participants	AI model	Classification	Clinicians’ vs AI	AI performance	Clinicians’ performance	Augmented performance
Piccolo et al.^9^	DEM-MIPS: ANN trained with 50 non-melanomas and 50 melanomas (training) Institutional (test)	Dataset: 341 (test) Training: 100	Test: 341	E	R	Y	N	2 participants - 1 trained dermatologist - 1 resident clinician	DEM-MIPS software (Digital Epi Microscopy Melanoma Image Processing Software; Biomips SRL, Siena, Italy).	Dichotomous:melanoma vs non-melanoma	Sn was comparable between experienced dermatologist and the computer. Sp. of the computer was lower.	Sn 92% Sp 74%	Expert: Sn 92%; Sp 99% Resident: Sn 69%; Sp 94%	
Friedman et al.^12^	Database acquired by Electro-Optical Sciences Inc for the development and testing of MelaFind Institutional (test and training)	Dataset: 990 Training: 75	Test: 99	I Δ	R	Y	Y	10 participants - 9 expert dermatologists - 1 dermatology nurse practitioner	Computer-vision system	Dichotomous: melanoma vs non-melanoma	For small lesions, AI had significantly higher Sn (*P* < 0.001). Sp was comparable.	Sn 98% (92–100) Sp 44% (29–59) Acc 62% (53–70) PPV 63% (56–70) NPV 96% (79–100)	Sn 71% (63–79) Sp 49% (40–58) Acc 47% (39–55) PPV 58% (51–64) NPV 63% (52–74)	
Dreiseitl et al.^28^	Institutional	Dataset: not specified Training: 1,311	Test: 3,021 (evaluated patients from institution)	I	P	B	N	1 expert dermatology 6 physicians, with the added decision-support system: -3 high experience -3 low experience	Matlab neural network model	Dichotomous: melanoma vs non-melanoma	The expert physician outperformed AI.	Sn 68% Sp 54% AUC 0.87 (0.82–0.92)	Expert Sp Sn 96% Sp 72%	Low experience: Sn 70%; Sp 81% High experience: Sn 74%; Sp 84%
Tenenhaus et al.^77^	Institutional	Dataset: 900 Training/validation: 100/80	Test: 227	I	R	B	N	5 senior dermatologists	KL–PLS-based classifier	Dichotomous: excision vs non-excision Multiclass: melanoma, dysplastic or benign lesion	Comparable	Sn 95% Sp 60%	Sn 70.2% Sp 83.2% Therapeutic decision Sn 86.4% Sp 56.6%	
Ferris et al.^24^	Institutional	Dataset: not specified Training: 273	Test: 173	I	R	Y	N	30 participants: - 12 board-certified dermatologist - 10 dermatology resident - 8 dermatology physician assistant.	Not specified	Dichotomous: benign vs malignant	The classifier’s Sn to melanoma was higher (*p* < 0.001) and Sp was lower (*p* < 0.001) than clinicians.	Sn 96% Sp 42.5% AUC 0.818	Board-certified: Sn 64.7% Sp 65.4% Residents: Sn 70.4% Sp 59% Physician assistants: Sn 80.5% Sp 48.1%	
Tschandl et al.^25^	Institutional	Dataset: 298 Training: 298	Test: 50	I	R	N	N	27 last-year medical students without prior knowledge of dermoscopy to participate in a 1-h training session.	GoogLeNet Inception v3	Dichotomous: benign vs malignant	Comparable	Sn 90% (68–99) Sp 71% (51–87) AUC 0.91	Sn 86% (83–88) Sp 79% (74–83) AUC 0.85	
Yu et al.^26^	Institutional	Dataset: 724 Training: 364	Test: 364	I	R	Y	N	4 participants: -2 general physicians -2 experienced dermatologists	MatConvNet, modified VGG model with 16 layers	Dichotomous: acral melanoma, benign nevi	Comparable performance. For diagnostic Acc, both the CNN and expert group were higher than that of non-expert.	Subset A Sn: 92.57% (87.63–95.96) Sp: 75.39% (68.72–81.26) Acc: 83.51% (79.39–96.94) Subset B: Sn: 92.57% (87.63–95.99) Sp: 68.16% (60.79–74.91) Acc: 80.23% (75.77–84.04)	Subset A Expert: Sn 94.88% Sp 68.72% Acc 81.08% Non-dermatologist Sn 41.71% Sp 91.28% Acc 67.84% Subset B: Expert: Sn 98.29% Sp 65.36% Acc 81.64% Non-dermatologist Sn 48.00% Sp 77.10% Acc 62.71%	
Marchetti et al.^13^	Public: ISBI 2016 Melanoma Detection Challenge Dataset (ISIC Archive)	Dataset: 1,279 Training/validation: 900	Test: 379	I	R	B	N	8 dermatologists	Five top-ranked individual algorithms of the ISBI 2016 Challenge	Dichotomous: Melanoma vs non-melanoma	Dermatologist Sp was similar to the top challenge algorithm but lower than the best-performing fusion algorithm.	Top fusion computer algorithm: Sn 82% Sp 76% ROC 0.86	Sn 82% (68–98) Sp 59% (34–72) ROC 0.71 (0.61–0.76)	
Phillips et al.^27^	Public: not specified (training) Institutional (training and test)	Dataset: 1,550 Training: 858 images of 286 lesions from 92 patients.	Test: 1550	I	P	B	Y	Not specified	Deep Ensemble for Recognition of Malignancy	Dichotomous: Melanoma vs non-melanoma	Comparable	Sn 95% Sp 78.1%	Sn 95% Sp 69.9% ROC 0.778	
Tschandl et al.^30^	Public: ISIC 2018 + Institutional (Vienna Dermatologic Imaging Research Group (ViDIR)+ skin cancer practice of Cliff Rosendahl in Queensland + images from Turkey, New Zealand, Sweden, and Argentina)	Dataset: 11,210 Training/validation: 10,015	Test set: 1,511 (divided in 30-batches) ** 316 images from other centers to the test set (external data), specifically from Turkey, New Zealand, Sweden, and Argentina, to assure diversity of skin types	I	R	B	N	511 participants: -283 board-certified dermatologists -118 dermatology residents - 83 general practitioners	139 algorithms created; by 77 machine-learning labs. Top three machine-learning algorithms -MetaOptima Technology Inc -DAISYLab -Medical Image Analysis Group, Sun Yat-sen University	Multiclass	When comparing all human readers with all machine-learning algorithms, the algorithms achieved a mean of 2.01 (*p* < 0·0001) more correct diagnoses.	MetaOptima: Sn 88.5% (82.2–94.7) AUC 0.963 (0.953–0.973; *p* = 0.46) DAISYLab: Sn 85.6% (79.1–92.0) AUC: 0·971 (0.961 0.982; *p* = 0.05) Sun Yat-sen Uni: Sn 84.5% (78.5–90.5) AUC: 0.958 (0.945–0.972; *p* = 0.91)	Dermatologist: Sn 81.2% (66.1–96.3) All readers: Sn 79.2% (64.4–94.0) AUC: 0.958 (0.948–0.967)	
Brinker et al.^78^ (II)	Public: ISIC archive + HAM10,000 (training)	Dataset: 20,735 Training/validation: 12,378/1,259	Test: 100 dermoscopic images	I Δ	R	B	N	157 participants - 56.1% dermatologic residents - 43.9% board certified	ResNet50 CNN model	Dichotomous: Melanoma vs atypical nevi	AI outperforms dermatologists but not significant difference (*p* = 0.31).	Sn 74.1% Sp 86.5% (70.8-91.3)	Sn 74.1% (40.0-100) Sp 60% (21.3-91.3) ROC 0.671	
Brinker et al.^15^ (III)	Public: ISIC (training and test)	Dataset: - Training: 4,204	Test: 804 Test set: 134	I Δ	R	Y	N	144 participants: -52 board-certified dermatologists -92 junior dermatologists	ResNet50 CNN	Dichotomous: Melanoma vs nevi	CNN achieved a higher Sn and Sp. CNN was significantly superior to both junior and board-certified dermatologists (*p* < 0.001).	Sn 82.3% (78.3–85.7) Sp 77.9% (73.8–81.8)	Overall dermatologists: Sn 67.2% (62.6–71) Sp 62.2% (57.6-66.9) Board-certified dermatologists Sn 63.2% (58.7-68.1) Sp 65.2% (60.5-69.8)	
Hekler et al.^38^	Public: ISIC archive (training), HAM10000 (training and test)	Dataset: 11,444 Training: 11,394	Test: 300 Test set: 50	I	R	Y	N	112 dermatologists from 13 German clinics	ResNet50	Primary end-point: multiclass secondary end-point: dichotomous (benign vs malignant)	Combination of man and machine achieved an accuracy of 82.95%. This was 1.36% higher than the best of the two individual classifiers.	CNN Sn 86.1% (81.1–91.2) Sp 89.2% (83.6-94.7) Acc 81.59%	Physicians Sn 66% (59.1-72.9) Sp 62% (53.3-70.7) Acc 42.94%	Fusion method Sen 89% (84.4-93.6) Spe 84% (77.4–90.6) Acc 82.95%
Maron et al.^39^	Public: ISIC archive (training); HAM10000 (training and test)	Dataset: not specified Training: 11,444	Test: 300	I	R	B	N	112 dermatologists of 13 German university hospitals	ResNet50	Primary end-point: dichotomous (benign vs malignant) Secondary end-point: multiclass (5 diagnostic categories)	CNN significantly outperformed the dermatologists (*p* < 0.001) Multiclass classification: outperformance (*p* < 0.001) was achieved except for BCC (on-par performance).	Dichotomous: Sn 74.4% (67.0–81.8) Sp 91.3% (85.5–97.1) Multiclass: Sn 56.5% (42.8–70.2) Sp 98.8%	Dichotomous: Sn 74.4% (67.0–81.8) Sp 59.8% (49.8–69.8) Multiclass: Sn 56.5% (42.8–70.2) Sp 89.2% (85.0–93.3)	
Maron et al.^16^	Public ISIC, HAM 10000 (training and test)	Dataset: not specified Training: 4,894	Test set: 1,200 Test: 100×12	I Δ	R	Y	N	-12 dermatologists from 9 German university hospitals	CNN	Dichotomous: melanoma vs nevi	CNN had higher Sn, Sp and Acc than dermatologists. Mean Sn and Acc increased significantly (*p* = 0.003 and *p* = 0.002, respectively) with AI support. Sp did not deteriorate substantially.	Sn 84.7% (81.9–87.6) Sp 79.1% (74.8–83.4) Acc: 81.9% (79.7–84.2)	Sn 59.4% (53.3–65.5) Sp 70.6% (62.3–78.9) Acc 65.0% (62.3–67.6)	Sn 74.6% (69.9–79.3) Sp 72.4% (66.2–78.6) Acc 73.6% (70.9–76.3)
Lee et al.^17^	Institutional (pigmentary lesions collected from 2014 to 2019 at the Department of Dermatology, Severance Hospital, Seoul, Korea) - training and test	Dataset: 1,072 Training: 872	Test: 200	I Δ	R	Y	Y	60 participants - 20 board-certified dermatologists - 20 dermatology residents - 20 general physicians	ALMnet (ResNet with 50 residual layers)	Stage I: dichotomous: melanoma (acral lentiginous melanoma) vs nevi Stage II: additional clinical information. Stage III: dermatologists + ALMnet diagnosis	ALMnet outperforms clinicians	Test set-200: Sn 96% (82.4–95.1) Sp 95% (88.7–98.4) Acc 92.5% (87.9–95.7) AUC 0.976 (0.974–0.978) Human-set Stage-I: Sn 92% (80.8–97.8) Sp 96% (86.3–99.5) Acc 94% (87.4–97.8)	Stage I Sn 79.9% (76.2–83.5) Sp 69.5% (65.1–73.8) Acc 74.7% (72.6–76.8) Stage II: Sn 81.5% (77.7–85.2) Sp 76.4% (72.5–80.4), Acc: 79.0% (76.7–81.2),	Stage III: Sn 88.7% (86.0–91.5) Sp 85% (82.7–87.3), Acc 86.9% (85.3–88.4) Significant improvement in participants’ performances, emphasized in the relatively inexperienced groups.
Marchetti et al.^18^	Public ISIC 2017 (training and test)	Dataset: 2,750 Training/validation: 2,000/150	Test: 600 Test set: 150	I	R	N	N	17 participants: -8 dermatologists -9 dermatology residents	23 algorithms	Dichotomous: melanoma vs non-melanoma	ROC of the top-ranked algorithm in melanoma classification was greater than the overall ROC in classification and management of dermatologists and residents (*p* < 0.001 for all comparisons). At the dermatologists’ overall Sn, algorithm had a higher Sp (*p* = 0.001).	ROC top algorithm: 0.868 Sn 76% Sp 85% Management decision: Sn 89% Sp 61%	Dermatologists Sn 76% (71.5–80.1) Sp 72.6% (69.4–75.7) ROC 0.74 (0.72–0.77) Residents Sn 56% (51.3–60.6), Sp 76.3% (73.4–79.1) ROC 0.66 (0.6–0.69)	Resident: Sn from 56% to 72.9% Sp from 76.3% to 72.6%. Dermatologist Sn from 76% to 80.8% Sp from 72.6% to 72.8%
Wang et al.^34^	Institutional: Images collected from Department of Dermatology, Peking Union Medical College Hospital, between 2016 and 2018	Dataset: -Data set I/multiclass: 7,192 (tumors) -Data set II: 3,115 (inflammatory) Training/validation/test: 8:1:1 ratio	Test set: 130 total 70 multiclass	I Δ	R	B	N	164 dermatologists with dermoscopic training	GoogLeNet Inception v3 using the ImageNet dataset.	Multiclass	Comparable. There was no significant difference in Kappa coefficients (*P* > 0.05).	BCC Sn 80%, Sp 100% Nevus Sn 80%, Sp 84% SK Sn 85%, Sp 94% Other lesions Sn 75%, Sp 94% Acc: 81.49% ± 0.88	BCC Sn 77%; Sp 96.2%; AUC 0.972 ± 0.011 Nevus Sn 80.7%; Sp 89.7%. AUC 0.952 ± 0.014 SK Sn 62.4%; Sp 97.6%; AUC 0.933 ± 0.014 Other lesions: Sn 93.9%; Sp 87.5% AUC 0.965 ± 0.005	
Lucius et al.^35^	Public: HAM10000 dataset ISIC archive (training and test)	Dataset: 10,015 Training/validation: 8,313	Test: 1,702	I Δ	R	Y	N	41 general practitioners	ResNet34 ResNet50 ResNet101 SEResNet50 VGG16 VGG19 EfficientNetB5 MobileNet	Multiclass Second challenge: diagnosis with time constraint (45s per image)	EfficientNetB5 global Acc significantly outperformed physicians. With assistance, the global Acc increased by 25.13%.	Global Acc 76.3% ± 2.79 Second challenge: EfficientNetB5 Acc 77.14%, error rate 22.86%	First challenge: Acc 27.74%; error rate 72.26% Second challenge: Acc 17.29%; error rate 82.71%	General practitioners + AI: Acc 42.43%, Error rate 57.57%
Minagawa et al.^31^	Public: ISIC 2017, HAM10000, BCN20000 dataset (training and test) Institutional: Shinshu set (training and test)	Training: 12,254 ISIC + 594 Shinshu set.	Test: 100 (50 public + 50 Shinshu)	I Δ	R	B	N	30 Japanese dermatologists: - included 20 board-certified dermatologists	Inception-ResNet-v2	Multiclass	The Sp of the algorithm at the dermatologists’ mean Sn was significantly higher than human readers (*p* < 0.001).	At human mean Sn: Shinshu set: Sn: 85.3%, Sp 96.2% Acc: 94% ISIC: Sn 60.8%, Sp 100% Acc: 94%	Shinshu set -All: Sn 85.3%; Sp 92.2% -Board-certified: Sn 87.1%; Sp 92.9% -Other: Sn 81.7%; Sp 90.8% -Acc: 88% (87.1–90.7) ISIC set -All: Sn 60.8%; Sp 92.6% -Board-certified: Sn 62.7%; Sp 93.1% -Other: Sn 57.1%; Sp 91.5% -Acc: 77% (75–79.7)	
Fink et al.^19^	Public: Moleanalyzer-Pro; FotoFinder Systems GmbH, pre-trained architecture additionally trained with >120.000 dermoscopic images and labels. (training) Institutional (Heidelberg, Gottingen), and Munich. (test)	Dataset: 129,487 Training: 115,099	Test: 72	E	R	B	N	11 dermatologists, level of experience in dermoscopy: - Beginner: <2 years - Skilled: 2–5 years - Expert: ≥5 years	GoogleNet Inception_v4 architecture	Dichotomous: Combined nevus vs melanoma Augmented performance: Scenario 1: CNN used to verify a diagnosis of malignancy. Scenario 2: CNN used to verify a diagnosis of benignity.	The tested CNN classified more accurately combined naevi and melanomas, in comparison with trained dermatologists	Sn: 97.1% (82.7–99.6, *p* = 0.092) Sp: 78.8% (62.8–89.1.3, *p* = 0.256) OR: 34 (4.8–239)	Average dermatologist: Sn: 90.6% (84.1–94.7) Sp: 71% (62.6–78.1) OR: 24; 11.6–48.4, *p* = 0.1114	Scenario 1: Sp from 71% to 90.3%; Sn from 90.6% to 88.7%. Scenario 2: Sn would increase to 99.9%. However, would be accompanied by a non-ignorable loss of Sp.
Tschandl et al.^32^	Public: HAM10000 dataset Test set of the ISIC 2018 challenge	Training: not specified	Test: 1,511 928 from Medical University of Vienna, 267 from Cliff Rosendahl in Queensland, 316 images from other centers in Turkey (n = 117), New Zealand (n = 87), Sweden (n = 92) and Argentina (n = 20),	I	R	B	N	302 raters from 41 countries -169 board-certified dermatologists -77 dermatology residents -38 general practitioners.	ResNet34	Multiclass	Accuracy was superior for CNN	CNN Mean recall for all disease categories: 77.7% (70.3% to 85.1%) Acc 80.3%	Acc: 63.6%	Multiclass probabilities: improved the Acc of human raters from 63.6% to 77%. Prediction of malignancy: no improvement observed.
Tognetti et al.^20^	Public: ISIC archive, iDScore dataset (collected from 8 European centers)	Pre-training: 20,735 Training/validation: 630/135	Test: 214	I	R	Y	Y	111 dermatologists with different levels of experience in dermoscopy. Aware of clinical data	DCNN_aMSL (modified version of the ResNet50) iDCNN_aMSL (images + clinical data)	Dichotomous: Melanoma vs atypical nevus	The average dermatologists showed performance on the testing set far below both DCNNs (*p* < 0.05)	DCNN Sn: 89.2% (80.8–94.7) Sp: 65.7% (61.3–68.6) ROC 77.5% (71.0–83.9) AUC 0.866 (0.813–0.92) iDCNN Sn: 86.5% (77.9–92.6) Sp: 73.6% (69.0–76.8) ROC 80% (73.8–86.3) AUC 0.903 (0.863–0.944)	Sn: 77% (65.8–86.0) Sp: 61.4% (52.8–69.5) ROC 69.2% (61.9–76.6) ROC level I-II (less experienced): 68.2% (59.976.5) ROC level III-IV (more experienced): 71.8% (64.379.3)	
Winkler et al.^29^ (I)	Public: HAM10000 dataset (training) Institutional (test)	Training: CNN1: >150,000 dermoscopic images (Moleanalyzer-Pro®). CNN2: images from the HAM10000 dataset	Test: 236	E	R	B	N	26 dermatologists with three different levels of experience	CNN1: GoogleNet Inception v4 (Moleanalyzer-Pro®, Foto-Finder Systems GmbH, Bad Birnbach, Germany) CNN2: Resnet34 architecture	Dichotomous: Melanoma vs nevus	The tested CNN could not replace the strategy of Sequential digital dermoscopy (SDD). Diagnostic sensitivities were significantly higher in follow-up images than in baseline images for both CNN (*p* < 0.05). Comparing the number of baseline quartets correctly classified, both CNN were outperformed by dermatologists (*p* < 0.001).	CNN1: Baseline Acc 15.3% Sn 25.4% (16.1-37.8) Sp 92.7% (87.8–95.7) ROC 69.6% (62.1-77.1) Follow-up quartets Acc 28.8% Sn 44.1% (32.2–56.7) Sp 92.7% (87.8–95.7) ROC 81.7% (75.7–87.6) CNN2: Baseline Acc 13.6% Sn 28.8% (18.8-41.4) Sp 75.7% (68.9-81.4) ROC 58.7% (50.5-66.9) Follow-up quartets Acc 20.3% Sn 49.2% (36.8-61.6) Sp 75.7% (68.9-81.4) ROC 69.8% (62.2- 77.4)	Baseline Acc 40.7% Sn 66.1% Sp 55.4%	
Winkler et al.^40^ (II)	Public (training) Institutional: 30 cases of difficult-to-diagnose skin lesions (test)	Training: CNN further trained with > 150,000 labeled dermatoscopic images.	Test: 30	E	R	Y	Y	120 dermatologists during a live conference.	Binary: GoogleNet Inception v4 architecture (Moleanalyzer-Pro®, Foto-Finder Systems GmbH, Bad Birnbach, Germany)	Dichotomous: benign vs malignant Multiclass	The diagnostic accuracy of collective human intelligence (CoHI) was superior to that of individual dermatologists (*P* < 0.001) in multiclass evaluation, with the accuracy of the latter comparable to multiclass CNN. CoHI outperformed individuals and CNN in a demanding skin lesion classification task.	Binary Acc 70.0% (52.1–83.3) Sn 70.6% (46.9–86.7) Sp 69.2% (42.4–87) ROC 0.765 (0.595–0.935) Multiclass Acc 62.5%	Binary Sn 77.7% (75.3–80.2) Sp 73.0% (70.6–75.4) Acc 75.7% (73.8–77.5) Multiclass Acc 64.6% (61.6–67.6)	
Haenssle et al.^21^ (II)	Institutional: University of Heidelberg; Hospital Thalkirchner Street, Munich; Medical University of Graz; Aristotle University, Thessaloniki; clinic of Dermatology, Konstanz. (test) Additional test: ‘Australian data set’ (240) ‘ISIC2018 data set’:1511 ‘MSK-1 data set’: 1100 ‘Prospective data set’ a real-world dermoscopic data set of 1981 lesions	Training: CNN pre-trained (Moleanalyzer-Pro®)	Test: 100 Additional external test set of 4832 images	E	R	B	Y	64 Dermatologists with 3 different self-reported levels of experience: -Beginner (*n* = 9) -Skilled (*n* = 20) -Expert (*n* = 30) -Unknown (*n* = 5)	Moleanalyzer-Pro, FotoFinder Systems, Bad Birnbach, Germany (modified architecture of Google’s Inception_v4)	Dichotomous: -Malignant/benign -Excision or treatment/ follow-up or no action Level I: dermoscopy only Level II: dermoscopy, clinical close-up images, information	Dermatologists of all training levels were outperformed by the CNN (all *p* < 0.001).	Sn 96.2% (87.0-98.9) Sp 68.8% (54.7-80.1) AUC 92.9 (88.0-97.8)	level I (dermoscopy) All: Sn 77.1% (74.0-80.2) Sp 69.5% (66.3-72.7) Acc 73.4% Beginner: Sn 69.4%; Sp 67.6%; Acc 68.6% Skilled: Sn 78.0%; Sp 67.8%; Acc 73,1% Expert: Sn 80.6%; Sp 72.2%; Acc 76.6% Unknown: Sn 70.1%; Sp 70.5%; Acc 70.3%. Level II (dermoscopy + close-up + textual case information): All: Sn 84.2% (82.2–86.2) Sp 69.4% (66.0–72.8) Acc 77.1% Beginner: Sn 82.9%; Sp 63.0%; Acc 73.3% Skilled: Sn 84.3%; Sp 69.3%; Acc 77,1% Expert: Sn 85.1%; Sp 72.6%; Acc 9.1% Unknown: Sn 80.8%; Sp 61.7%; Acc 71.6%.	
Zhu et al.^36^	Institutional Peking Union Medical College Hospital in China	Training: 13,603	Test: 200	I	R	B	N	280 board-certificated dermatologists	Google’s EfficientNet-b4 with pre-trained weights on the 2015 ImageNet dataset	Multiclass	Comparable performance.	Sn 83.50% Sp 94.07% Acc 92.75%	Sn 68.51% Sp 95.50% Acc 92.13%	
-Pham et al.^23^	Public ISIC 2019. MClass-D dataset of Titus J. Brinker et al.	Dataset: 17,302. (4503 melanoma and 12,799 nevus) Training: 13,842 Validation: 1,730	Test: 1730, 450 melanoma and 1280 nevus	I Δ	R	B	N	157 dermatologists at different German university hospitals	wInceptionV314, ResNet5015, DenseNet16916. New deep architecture with introduction of custom loss function, custom mini-batch logic, and optimized fully connected layers.	Dichotomous: melanoma vs nevus	BLF (best model) surpasses the performance of every dermatologist.	AUC 94.4% Sn 85% Sp 95%	AUC 67.1% Sn 74.1% Sp 60.0%	
Zhen Yu et al.^23^	Public HAM-10000 dataset (training) Institutional (training and test)	Dataset: 179 serial dermoscopic images from 122 patients, total 730 images. Training: 90% Validation 10%	Test: not specified	I Δ	R	Y	Y	12 dermatologists - 7 experienced dermatologists -5 registrars	ResNet-34	Dichotomous: benign vs. malignant	The model achieved higher diagnostic Acc than clinicians and provided an earlier diagnosis of melanoma (60.7% vs. 32.7%) on the first follow-up images.	Acc 63.69% Sn 60.67% Sp 66.67%	Overall clinicians Acc 54.33% Sn 61.99% Sp 46.76% Dermatologists Acc 56.19% Sn 61.80% Sp 50.63% Registrars Acc 51.73% Sn 62.25% Sp 41.33%	
Van Molle et al.^37^	Public: HAM10000 (training and test)	Training/validation not specified.	Test: 30	I Δ	R	N	N	22 professional dermatologists	ResNet50 model	Multiclass	Individual dermatologists scored similar to CNN, with the average dermatologist scoring slightly better.	Acc 46% Sn 50% Sp 88% ROC 0.69	Mean Acc 55% Sn 68% Sp 73% ROC 0.70	
Combalia et al.^33^	Public HAM10000 and BCN20000 (training and test) Turkey, New Zealand, Sweden, and Argentina (test)	Training: 25,331 Validation: 100 (HAM10000)	Test: 8,238 from BCN, HAM, Turkey, New Zealand, Sweden, and Argentina	I Δ	R	B	Y	18 expert dermatologists	EfficientNet and ResNet	Multiclass	Algorithms performed better than experts in most categories, except for AK (similar accuracy on average) and images from categories not included in training data (p < 0.0001).	Top Acc: 63.6% Mean Acc 50% Mean Acc + metadata: 56% Acc: AK 83%, BCC 91%, BKL 43%, DF 73%, MEL 70%, Nevus 70%, NT 1%, CC 62%, VASC 79%.	Acc: AK 43%, BCC 70%, BKL 48%, DF 50%, MEL 62%, Nevus 56%, NT 26%, SCC 65%, VASC 83%.	

*HP* histopathology confirmation, *I/E* internal/external test set, *P* prospective, *R* retrospective, *B* both (a subset of lesions were biopsy proven and a subset based on clinical/consensus diagnosis), *CD* clinical data (metadata) available, *CNN* convolutional neural network, *DCNN* deep convolutional neural network, *AK* actinic keratosis, *BCC* basal cell carcinoma, *BKL* benign keratosis, *SK* seborrheic keratosis, *DF* dermatofibroma, *MEL* melanoma, *NT* not trained, *SCC* squamous cell carcinoma, *VASC* vascular lesion, *Sn* sensitivity, *Sp* specificity, *Acc* accuracy, *NPV* negative predictive value, *PPV* positive predictive value, *OR* odds ratio, *ROC* receiver operating characteristic curve, *AI* artificial intelligence. Δ hold-out dataset.

**Table 2:** Included studies general characteristics, dataset used, and performance evaluating clinical images

Author	Database	Training set	Test set	I/E	Design	HP	CD	Participants	IA	Classification	Clinicians’ vs IA	IA performance	Clinicians’ performance	Augmented performance
Chang et al.^47^	Institutional: Kaohsiung Medical University	Dataset: 24,178 Training/validation: not specified	Test: 769	I	R	Y	N	25 dermatologists	CADx system	3-class: Malignant or benign or indeterminate	Comparable	Sn 85.63% Sp 87.65% Acc 90.64% ROC 0.949	Sn 83.33% Sp 85.88% Acc 85.31%	
Han et al.^42^	Public: Training: Asan dataset, MED-NODE dataset, and atlas site images	Dataset: 598,854 Training: 19,398 Validation: portion of the Asan, Hallym and Edinburgh datasets.	Test: 480 images (260 images Asan test, 220 images Edinburgh)	I	R	B	N	16 dermatologists: -10 professors -6 clinicians	Microsoft ResNet-152 model	Dichotomous: Benign vs malignant	Comparable	Asan dataset: Sn 86.4% ± 3.5% Sp 85.5% ±3.2% AUC 0.91 ± 0.01 Edinburgh: Sn 85.1% ± 2.2% Sp 81.3% ± 2.9% AUC 0.89 ± 0.01		
Fujisawa et al.^43^	Institutional: University of Tsukuba Hospital from 2003 to 2016 (training and test)	dataset: 6,009 training/validation: 4,867	Test: 1,142	IΔ	R	B	N	22 dermatologists: -13 board-certified -9 trainees	GoogLeNet DCNN model	Dichotomous: Benign vs malignant	DCNN achieved greater accuracy (P< .0001).	Sn 96.3% Sp 89.5% Acc 76.5%	Acc board-certified 85.3% ± 3.7% Acc trainees 74.4% ± 6.8%	
Han et al.^44^	Public: MED-NODE data set, Seven-Point Checklist Dermatology data set (training) Institutional: Asan Medical Center Department of Dermatology, Hallym National University Department of Plastic Surgery, Chonnam University Department of Plastic Surger (training and test set)	Dataset: Training/validation: 1,106,886/2,844	Test: 325	I	R	Y	N	119 clinicians: -13 board-certified dermatologists -34 dermatology residents -20 non-dermatologic physicians -52 general public with no medical background	Blob detector training using faster-RCNN20, a fine image selector and the disease classifier training using CNNs (SENet, SE-ResNeXt-50, and SE-ResNet-50).	Dichotomous: Benign vs malignant	Comparable	AUC: 91.9 Sn 98.2% Sp 77.9%	Dermatologists ROC: 0.90 Non-dermatologist physicians ROC: 0.725 (Sn and Sp for each one not specified) Overall: Sn 95.0% Sp 72.1%	
Zhao et al.^48^	Institutional: XiangyaDerm, which was collected from Xiangya Hospital	Dataset: 150,223 Training/validation: 4,500	Test: 60	I	R	Y	N	20 dermatologists	Xception architecture	3 risk classification: low risk, high risk, and dangerous	Classifier outperforms dermatologists	Acc 82.7% Benign: Sn 93%, Sp 88% Low degree: Sn 85%, Sp 85% High degree: Sn 86%, Sp 91% AUC: - Low-risk: 0.959 -High-risk: 0.919 - Dangerous: 0.947	Sn: - Low-risk: 61% - High-risk: 49.5% - Dangerous: 64% Sp - Low-risk: 4.9% -High-risk: 29% - Dangerous: 29%	
Han et al.^52^	Public: Asan Medical Center and images from websites (training) Institutional: Department of Dermatology, Severance Hospital, Seoul, Korea (test set)	Dataset: - Dataset A (Dichotomous): 40,331 - Dataset B (Multiclass): 39,721 Training: 1,106,886 images	Test: 1,320	E	R	Y	N	65 attending physicians (dichotomous) 44 dermatologists 5.7 ± 5.2 years of experience (multiclass)	Disease classifier (SENet and SE-ResNeXt-50) was trained with the help of a region-based CNN (faster RCNN)	Dichotomous: benign or malignant Multiclass: diagnosis	First clinical impression of physicians was superior to those of the algorithm Multiclass classification was comparable.	Dichotomous: AUC 0.863 (0.852–0.875) Sn 62.7% (59.9–65.1) Sp 90.0% (89.4–90.6) PPV 45.4% (43.7–47.3) NPV 94.8% (94.4–95.2) Multiclass: Sn 66.9% (57.7–76.0) Sp 87.4% (82.5–92.2)	Dichotomous: Sn 70.2% Sp 95.6% PPV 68.1% NPV 96.0% Multiclass: Sn 65.8% (55.7–75.9) Sp 85.7% (82.4–88.9)	
Huang et al.^45^	Institutional: Xiangya Hospital, Central South University,	Dataset: 3,299 Training: 2,474	Test: 825 Additional test set: 116	IΔ	R	Y	N	21 participants: -8 expert dermatologists -13 general dermatologists	4 CNN networks: InceptionV3, Inception-ResNetV2, DenseNet121, and ResNet50	Dichotomous: BCC vs SK	InceptionResNetV2 model outperformed general dermatologists and was comparable to expert dermatologists.	PPV 89.7% NPV 10.3% AUC 0.937	PPV 73.2% NPV 21.5%	
Han et al.^53^ (I)	Public: ASAN, Web, MED-NODE, images from websites (training). Edinburgh dataset (validation) Institutional: SNU datasets (validation and test) SNU dataset consisted of data from three university hospitals (Seoul National University Bundang Hospital, Inje University Sanggye Paik Hospital, and Hallym University Dongtan Hospital)	Dataset: 224,181 Training: 220,680, 174 disease classes Validation: SNU dataset: 2,201 images of 134 disorders Edinburgh dataset: 1,300 images of 10 tumorous skin diseases.	Test: 240 images from SNU dataset	E	R	B	N	70 participants: - 21 dermatologists - 26 dermatology residents - 23 non-medical professionals	Not specified	Dichotomous: melanoma vs nevus and suggesting treatment option Multi-class classification of 134 skin disorders	Dichotomous: algorithm showed similar performance as dermatology residents but slightly lower than dermatologists	SNU AUC 0.937 ± 0.004 Edinburgh AUC 0.928 ± 0.002 Multiclass: mean top 1, 3, and 5 accuracies: 44.8 ± 1.2%, 69.0 ± 0.9%, and 78.1 ± 0.3%	Dermatologists Sn 77.4% ± 10.7 Sp 92.9% ± 2.4 AUC 0.66 ± 0.08 Non-medical professionals Sn 47.6 ± 33.1%	Sn and Sp of clinicians were improved by 12.1% (p < 0.0001) and 1.1% (p < 0.0001), respectively. Non-medical professionals improved Sn from 47.6 ± 33.1% to 87.5 ± 17.2% (p < 0.0001) without loss in Sp.
Jinnai et al.^54^	Institutional: Department Dermatologic Oncology in the National Cancer Center Hospital (training and test)	Dataset: 5846 Training/validation: 4732 images.	Test: 200 images	IΔ	R	B	N	20 dermatologists: -10 board-certified dermatologists (BCDs) - 10 dermatologic trainees (TRNs)	Faster, region-based CNN (FRCNN)	-Dichotomous: benign vs malignant -Multiclass: Six-class classification	Accuracy of FRCNN was significantly better than that of the dermatologists (p < 0.00001)	Dichotomous: -Acc: 91.5% -Sn: 83.3% -Sp: 94.5% Multiclass: -Acc: 86.2% -VPN 5.5% -VPP 84.7%	Dichotomous: BCDs: Acc 86.6%, Sn 86.3%, Sp 86.6%, TRNs: Acc: 85.3% Sn 83.5%; Sp 85.9% Multiclass: Acc: BCDs 79.5%; TRNs 75.1%	
Polesie et al.^46^	Institutional: department of Dermatology at Sahlgrenska University Hospital	Dataset: 1,551. 819 Melanoma in situ and 732 invasive melanomas. Training/validation: 1,051/200	Test: 300 images	IΔ	R	Y	N	7 dermatologists: -1 resident physician -6 board-certified dermatologists	De novo CNN	Dichotomous: in situ vs invasive melanoma	CNN was outperformed by dermatologists.	AUC 0.72 (95% CI 0.66–0.78)	AUC: 0.81 (95% CI 0.76–0.86	
Pangti et al.^49^	Public: public archives (http://www.hellenicdermatlas.com/en and http://www.danderm.dk/atlas, dermatologists across India.) (training) Institutional	Training/validation: 17,784 images, 40 skin diseases.	Test: 100 images, 58 biopsy-proven BCC, 42 facial non-BCC lesions.	E	R	B	N	50 participants: - 36 dermatologists - 14 non-dermatologists: 5 surgeons and 9 general physicians	DenseNet-161 Tensorflow	Multiclass	Sn and Acc of the app were significantly higher than both dermatologists (*P* < 0.0001) and non-dermatologists (*P* < 0.0001). The Sp was comparable (*P* = 0.07).	AUC 0.933 Sn 80.24 ± 3.11% Sp 91.57 ± 2.66% Acc 84.97 ± 2.45%	BCC diagnosis -Dermatologists: Sn 45.98% ± 21.21 Sp 96.03% ± 6.52 Acc 65% ± 11.7 -Non-dermatologists: Sn 10.71% ±10.53 Sp 98.47% ±3.19 Acc 47.57% ± 6.32	
Agarwala et al.^50^	Public: Triage tool www.triage.com free online system composed of four CNN models (training) Institutional (test)	Training: > 200,000 images, > 500 skin conditions	Test: 353 images	E	R	B	Y	21 US board-certified dermatologists	Triage algorithm	Multiclass	Accuracy of the dermatologist’s was better than the AI accuracy	Acc 63.3%; 95% CI 58.0–68.4%)	Acc: 69.1% (95% CI 63.7–74.1)	
Kim et al.^51^	Public Pre-trained algorithm Institutional: Department of Dermatology, Asan Medical Center, Seoul National University, Bundang Hospital (Test)	Training: 721,749 images, 178 disease classes	Test: 285 images	E	P	B	N	-10 attending physicians (11.4 ± 8.8 years’ experience after board certification) -11 dermatology trainees -7 intern doctors	Model Dermatology; https://modelderm.com	Multiclass	There was no direct comparison between AI and clinicians	Top-1 of the algorithm Sn 52.2% Sp 93.4% Acc 53.5% Top-2 of the algorithm Sn 69.6% Sp 78.5% Acc 66.0% Top-3 of the algorithm Sn 78.3% Sp 66.1% Acc 70.8%	Top-1 Dermatologist Sn 79.3% Sp 90.2% Acc 61.8% Trainees Sn 65.5% Sp 81.3% Acc 46.5% Top-2 Dermatologist Sn 86.2% Sp 82.1% Acc 69.4% Trainees Sn 93.1% Sp 51.8% Acc 54.2% Top-3 Dermatologist Sn 86.2% Sp 79.5% Acc 71.5% Trainees Sn 93,1% Sp 49.1% Acc 54.9%	Top-1/Top-2/Top-3 accuracies after assistance were significantly higher than those before assistance AI augmented the diagnostic accuracy of trainee doctors
Ba. et al.^41^	Institutional: Chinese PLA General Hospital & Medical School	Dataset: 29,280 Training/validation: 25,773. 10 categories of cutaneous tumors	Test: 400 from 2107 images dataset.	I Δ	R	Y	N	18 board-certified dermatologists, with different levels of experience	EfficientNet-B3	Dichotomous: malignant vs benign	CNN had higher Acc than un-assisted dermatologists. CNN-assisted dermatologists achieved a higher Acc and kappa (*p* < 0.001) than unassisted dermatologists Dermatologists with less experience benefited more from CNN assistance.	Multiclass Acc 78.45% Dichotomous Sn 83.21% Sp 91.3% (85.5-97.1)	Multiclass Acc 62.78% Dichotomous Sn 83.21% Sp 80.92%	Multiclass Acc: 76.60% vs. 62.78%, *p* < 0.001; kappa 0.74 vs. 0.59, *p* < 0.001 Dichotomous Sn 89.56% vs. 83.21%, *p* < 0.001 Sp 87.90% vs. 80.92%, *p* < 0.001

*HP* histopathology confirmation, *I/E* internal/external test set, *P* prospective, *R* retrospective, *B* both (a subset of lesions were biopsy proven and a subset based on clinical/consensus diagnosis), *CD* clinical data (metadata) available, *CNN* convolutional neural network, *DCNN* deep convolutional neural network, *AK* actinic keratosis, *BCC* basal cell carcinoma, *BKL* benign keratosis, *SK* seborrheic keratosis, *DF* dermatofibroma, *MEL* melanoma, *NT* not trained, *SCC* squamous cell carcinoma, *VASC* vascular lesion, *Sn* sensitivity, *Sp* specificity, *Acc* accuracy, *NPV* negative predictive value, *PPV* positive predictive value, *ROC* receiver operating characteristic curve, *AI* artificial intelligence. Δ hold-out dataset.

**Table 3:** Included studies general characteristics, dataset used, and performance evaluating both dermoscopic and clinical images

Author	Database	Dataset	Test	I/E	Design	HP	CD	Participants	IA model	Classification	Clinicians’ vs IA	IA performance (%, 95% CI)	Clinicians’ performance (%, 95% CI)	Augmented performance
Esteva et al.^55^	Public: ISIC, Edinburgh Dermofit Library, Stanford Hospital,	Dataset: 129,450 Training/validation: 127463	Test: 1942, 376 for comparison	I	R	Y	N	21 dermatologists	GoogleNet Inception v3	Malignant vs benign vs non-neoplastic Multiclass: 9-class	Comparable	Overall Acc 72.1% ± 0.9 9-class classification Acc 55.4% ± 1.7	Acc 65.78% 9-class classification Acc 54.15%	
Tschandl et al.^11^	Institutional databese from C.R., Australia (training and test). Medical University of Vienna, image database from C.R., and a convenience sample of rare diagnoses (test)	Training: 7895 dermoscopy 5,829 close-up Validation: 340 dermoscopy, 635 close-up	Test: 2,072 multiple sources.	I	R	Y	N	95 participants: Beginner ( < 3 y), intermediate (3-10 y), expert ( > 10 y).	CNN (combined model with outputs of 2 CNNs) InceptionV3 architecture30 ResNet50 network31	Dichotomous: benign vs malignant	Comparable	Sn 80.5% (79.0–82.1) Sp 53.5% (51.7-55.3)	Sn 77.6% (74.7-80.5) Sp 51.3% (48.4-54.3) mean AUC 0.695 (0.676–0.713): Beginners AUC 0.655; (0.626–0.684) Intermediate AUC 0.690; (0.657–0.722) Experts AUC: 0.741 (0.719– 0.763)	
Haenssle et al.^57^ (I)	Public: ISIC 2016. Institutional: Department of Dermatology, University of Heidelberg, Germany	Training/validation: not specified (ISIC)	Test: 100	E	R	N	Y	58 dermatologists: -17 Beginner <2, -11 Skilled 2–5 y -30 Expert >5 y	Google’s Inception v4 CNN architecture	Dichotomous: Melanoma vs nevus. Management decision (excision, short-term follow-up, no action).	CNN’s specificity was higher (82.5% vs 71.3%, *p* < 0.01). CNN ROC AUC (0.86 vs 0.79, *p* < 0.01).	Level I (dermoscopic images): Sn 86.6%. Sp 82.5% Level II (dermoscopy and clinical information) Sn 88.9% Sp 82.5%	Level I All: Sn 86.6% ( ± 9.3%); Sp 71.3% ( ± 11.2) ROC 0.79 Expert: Sn 89.0%, Sp 74.5% Skilled: Sn 85.9%, Sp 68.5% Beginner: Sn 82.9%, Sp 67.6% level-II All: Sn 88.9% ( ± 9.6%) Sp 75.7% ( ± 11.7, p < 0.05) ROC 0.82 Expert: Sn 89.5%, Sp 77.7% Skilled: Sn 90.0%, Sp 77.2% Beginner: Sn 86.6%, Sp 71.2%	
Brinker et al.^58^	Public ISIC 2017, HAM1000, MED-NODE database (training) Institutional (clinical images, test)	Dataset: 20,735 Training/validation: 12,378/1,359 dermoscopic images	Test: 100 clinical images	E	R	B	N	145 dermatologist -88 Junior physicians -16 Attendings -35 Senior physicians -3 Chief physicians	ResNet50	Dichotomous	Comparable	Sn 89.4% (55-100) Sp 68.2% (47.5-86.25)	All participants Sn 89.4% (55-100) Sp 64.4% (22.5–92.5) Junior Sn 88.9% Sp 64.7% ROC 0.768 Attendings Sn 92.8%, Sp 57.7%, ROC 0.753 Senior Sn 89.1%, Sp 66.3%, ROC 0.777 Chief Sn 91,70%, Sp 58.8%, ROC 0.753	
Li et al.^59^	Training: Chinese Skin Image Database (CSID), Youzhi AI software. Test: Institutional China-Japan Friendship Hospital.	Dataset: 1,438 patients Training: > 200,000 dermoscopic images	Test: 212 clinical, 106 dermoscopic	E	R	Y	N	11 participants: - 4 primary level - 4 intermediate - 3 dermoscopy experts.	Youzhi AI software (system version 2.2.5). GoogLeNet Inception v4 convolutional neural network architecture	Dichotomous: benign vs malignant	Comparable	Sn 74.84% ± 0.0149 Sp 92.96% ± 0.0052 Acc 85.85% Clinical images: Sn 71.1% ± 0.0169 Sp 90.6% ± 0.0107 Acc 83.02% Dermoscopic images: Sn 78.64% ± 0.0273 Sp 95.32% ± 0.0107 Acc 88.68%	AUC: 0.63 (0.55–0.71) Accuracy D Matched clinical and dermoscopy 86.02% Accuracy D Random 83.32% Clinical images: Acc 79.5% ± 0.0753 Dermoscopic images: Acc 89.62%	
Haenssle et al.^21^	Moleanalyzer Pro® (Training) Public MSK-1 dataset, ISIC-2018 (test set only for algorithm) Institutional (test)	Training: MSK-1 (1,100 images); ISIC-2018 (1511 images).	Test: 100 convenience sample collected between 2014 and 2019 MSK-1 dataset (1100) and ISIC-2018 dataset (1511) only for algorithm test.	E	R	B	Y	96 dermatologists: -17 beginners, <2 y -29 skilled 2–5 y -40 experts >5 y	Moleanalyzer Pro (Foto-Finder Systems GmbH, Bad Birnbach, Germany) CNN architecture based on Google’s Inception_v4,15	Dichotomous: malignant/premalignant vs benign. Management decision (treatment/ excision, no action, follow-up)	CNN and most dermatologists comparable performance.	Sn 95% (83.5%–98.6). Sp 76.7% (64.6%–85.6)	Level I dermoscopy: Sn 83.8%; Sp 77.6% Acc: Beginners 79.9% (77.7%–82.1%) Skilled 83.3% (80.1%–85.6%) Experts 86.9% (85.5%–88.3%). Level II dermoscopy + close-up + inf: Sn 90.6%; Sn 82.4% Acc: Beginners: 82.0% (79.3%–84.7%) Skilled: 85.4% (83.0%–87.8%) Experts: 88.5% (87.0%–90.0%)	
Willingham et al.^60^	Institutional Hawaii Pathologists’ Laboratory (training and test) Public ISIC dataset, MED-NODE, PH, DermNet, Asan and Hallym datasets (training)	Training: 14522 ISIC 539 Hawaii-based dermatologist image dataset.	Test: 50 (25 public, 25 institutional)	I	R	B	N	3 dermatologists	Google’s InceptionV3 network	Benign vs malignant Melanoma vs nonmelanoma.	Comparable.	AUC 0.948 Acc 68%	Acc: 64.7%	
Huang et al.^61^	Institutional Xiangya-Derm, (Chinese database, from 15 hospitals, that consists of over 150,000 images)	Data set: approximately 3000 images (six subtypes of skin diseases) Training: 2,400	Test: 600	IΔ	R	B	N	31 dermatologists: professors, senior attending doctors, young attending doctors, and medical students.	Xy-SkinNet, ResNet-101, ResNet-152 model	6-category common types of diseases.	AI-based classification accuracy exceeded the average accuracy of dermatologists	Top 3 Acc: 84.77%	Acc: 78.15%	

*HP* histopathology confirmation, *I/E* internal/external test set, *P* prospective, *R* retrospective, *B* both (a subset of lesions were biopsy proven and a subset based on clinical/consensus diagnosis), *CD* clinical data (metadata) available, *CNN* convolutional neural network, *DCNN* deep convolutional neural network, *AK* actinic keratosis, *BCC* basal cell carcinoma, *BKL* benign keratosis, *SK* seborrheic keratosis, *DF* dermatofibroma, *MEL* melanoma, *NT* not trained, *SCC* squamous cell carcinoma, *VASC* vascular lesion, *Sn* sensitivity, *Sp* specificity, *Acc* accuracy, *NPV* negative predictive value, *PPV* positive predictive value, *ROC* receiver operating characteristic curve, *AI* artificial intelligence. Δ hold-out dataset.

**Fig. 2:**
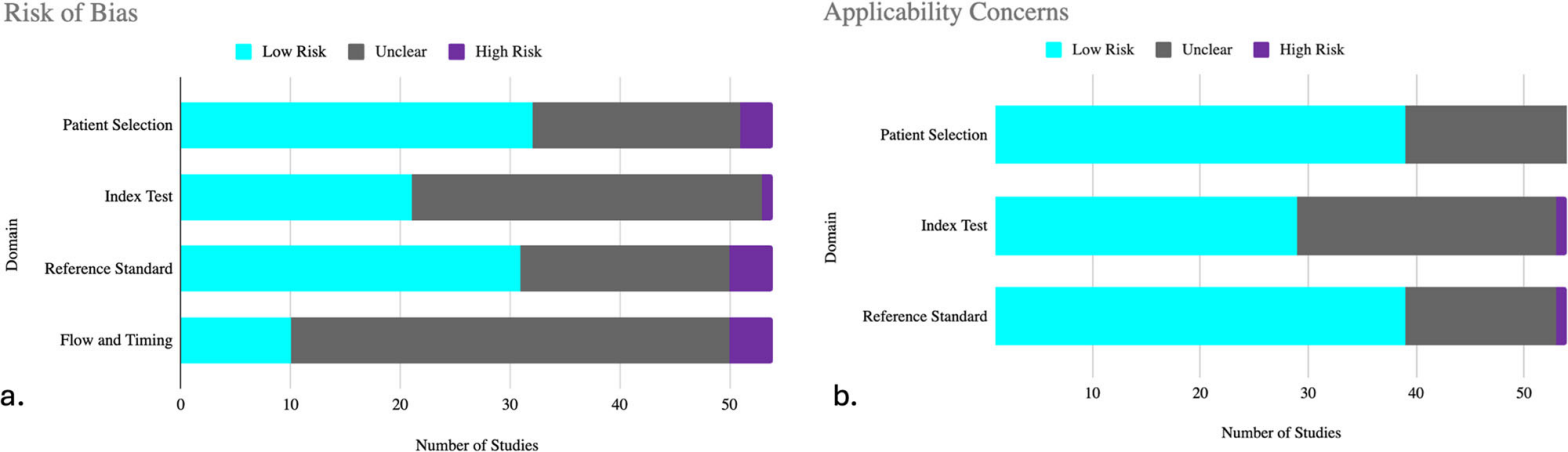
QUADAS-2 results of the assessment of risk of bias in the included studies. QUADAS-2 tool was used to assess the risk of bias in the included studies in terms of 4 domains (participants, index test, reference standard, and analysis). Low risk (cyan) refers to the number of studies that have a low risk of bias in the respective domain. Unclear (gray) refers to the number of studies that have an unclear risk of bias in the respective domain due to lack of information reported by the study. High risk (purple) refers to the number of studies that have a high risk of bias in the respective domain. **a**. Risk of Bias Assessment **b**. Applicability Concerns.

Regarding the risk of bias, most of the studies had an uncertain risk (58%), and 14 (26%) had a low risk of bias. Detail of QUADAS-2 score for each study included in the systematic review is in Fig. 2.

### Databases used

Only institutional or private databases were used in 20 articles (37.7%). In all, 16 articles (30.2%) used exclusively open-source data; the most commonly used databases were ‘ISIC’ and ‘HAM10000’^10,11^. Eighteen studies (33.9%) used a combination of institutional and public dataset. Twenty-two studies (41.5%) used only images of lesions confirmed with histopathology, while 27 (50.9%) included images diagnosed by expert consensus as the gold standard. Four studies (7.5%) did not specify a method of diagnosis confirmation. Fourteen studies (26.4%) used an external database for testing the algorithm, 39 studies (73.6%) tested with an internal dataset (Tables 1–3**)**.

### Study type and participants included

A total of 50 studies (94.3%) were retrospective and 3 (5.7%) were prospective. Twenty-seven studies (50.9%) included only specialists, in some cases detailing the level of expertise (expert dermatologists vs non-expert dermatologists). Twenty-three studies (43.3%) included dermatologists and other non-specialist clinicians (dermatology residents and/or generalists), and 3 studies (5.6%) included only generalists.

### Diagnosis included and metadata

Forty-three studies (81.1%) considered differential diagnosis between skin tumors only, while 10 (18.8%) also included inflammatory diagnosis or other pathologies (multiclass algorithms). Eighteen articles (33.9%) included clinical information on the patients (metadata), mainly age, sex, and lesion location.

### Artificial intelligence assistance

Of the total number of articles included in the review, 11 (20.7%) evaluated potential changes in diagnostic performance or therapeutic decisions of clinicians with AI assistance. Nine of 11 studies showed an improvement in global diagnostic performance when using AI collaboration, 6 of which showed a higher percentage of improvement in the generalists group.

### Diagnostic performance of artificial intelligence algorithm versus clinicians, from dermoscopic images of skin lesions

Thirty-one studies evaluated diagnostic performance with dermoscopic images **(**Table 1). In general, 61.2% (*n* = 19) of the studies showed a better performance of AI when compared to clinicians. A total of 29.0% (*n* = 9) resulted in a comparable performance, and in 9.7% (*n* = 3) specialists outperformed AI.

### Dichotomous classification (‘benign’ vs ‘malignant’)

Eighteen studies used AI with dichotomous classification (58.0%) as ‘benign’ vs ‘malignant’. In 61.1% AI outperformed clinicians (*n* = 11)^12–23^, being statistically significant in 54.5% of them^12,15,16,18,20,21^. A total of 27.7% showed comparable performance between AI and clinicians (*n* = 5)^9,24–27^. In all, 11.1% resulted in a better performance for clinicians in comparison to AI (*n* = 2)^28,29^, 1 of them showing statistical significance^29^. Five studies^16–19,28^ evaluated the collaboration between AI and clinicians (‘augmented intelligence’). All of them showed improved diagnostic accuracy when evaluating clinicians with the support of AI algorithms, being more relevant for less experienced clinicians. Statistical significance was demonstrated in two^16,17^.

### Multiclass and combined classification

Eight of the 31 studies used multiclass classification; in 4 of them, AI had a better performance^30–33^; in 3 studies the diagnostic accuracy was comparable^34–36^; and in 1 clinicians outperformed AI^37^. Two out of 8 studies evaluated AI-assistance, all of them showing improvement in diagnostic accuracy for human raters, with least experienced clinicians benefiting the most^32,35^. Five of the 31 dermoscopy studies developed both dichotomous and multiclass algorithms, 4 of them resulting in a better performance of AI over humans^38–41^.

### Diagnostic performance of artificial intelligence algorithms versus clinicians, using clinical images

A total of 14 AI articles evaluating CNN-based classification approaches that used clinical images only were included (Table 2). Of these, 42.8% (*n* = 6) showed a better performance of AI algorithms, 28.6% (*n* = 4) obtained comparable results, and in 28.6% (*n* = 4) clinicians outperformed AI.

### Dichotomous classification (‘benign’ vs ‘malignant’)

Six studies^42–46^ developed an AI algorithm with dichotomous outcomes, obtaining a performance comparable or superior to clinicians in 5 of them^42–45^. One study showed a better performance for clinicians^46^.

### Multiclass and combined classification

Five studies^47–51^ incorporated AI algorithms with multiclass classification. Zhao et al.^48^ and Pangti et al.^49^ obtained superior performance of AI algorithms, while Chang et al.^47^, showed comparable performance between AI and specialists. In one study, clinicians outperformed AI algorithm^50^.

Three studies^52–54^ with clinical images used both dichotomous and multiclass algorithms. Han et al.^53^ observed an improvement in diagnostic Sn and Sp with the assistance of the AI algorithm for both classifications, being statistically significant for less experienced clinicians.

### Diagnostic performance of artificial intelligence algorithms versus clinicians, from both clinical and dermoscopic images

Eight studies included clinical and dermoscopic images as part of their analysis^21,55–61^. Overall, 75% (*n* = 6) resulted in comparable performance, and 25% (*n* = 2) showed better performance for AI algorithms in comparison to clinicians. Only 1 study obtained statistical significance^57^.

### Dichotomous classification

Six studies applied dichotomous classification; Haenssle et al.^57^ being the only study obtaining a better performance for the AI algorithm over clinicians despite the incorporation of metadata. Five remaining studies showed a comparable performance between AI and clinicians.

### Multiclass and combined classification

Huang et al.^61^ classified into 6 categories, with AI being superior to specialists in average accuracy. Finally, Esteva et al.^55^ used two multiclass classifications, showing comparable performance between AI and clinicians in both.

### Meta-analysis

A total of 19 studies were included in the meta-analysis. Table 4 shows the summary estimates calculated to compare performance between AI and clinicians with different levels of experience.

**Table 4:** Meta-analysis results, summary estimates of sensitivity, specificity, and likelihood ratio according to subgroups

	Measure	Sensitivity	Specificity	LR +	LR -
Overall clinicians (*n* = 19 studies)	Summary estimate AI	87.0% (95% CI 81.7–90.9%)	77.1% (95% CI 69.8–83.0%)	3.79 (95% CI 2.89–4.97)	0.17 (95% CI 0.12–0.23)
	Summary estimate overall clinicians	79.8% (95% CI 73.2–85.1%)	73.6% (95% CI 66.5–79.6%)	3.02 (95% CI 2.33 – 3.91)	0.27 (95% CI 0.20–0.37)
Generalists (*n* = 5 studies)	Summary estimate AI	92.5% (95% CI 88.9–94.9%)	66.5% (95% CI 56.7–75.0%)	2.76 (95% CI 2.10-3.61)	0.11 (95% CI 0.07-0.16)
	Generalist	64.6% (95% CI 47.1–78.9%)	72.8% (95% CI 56.7–84.5%)	2.37 (95% CI 1.63-3.46)	0.48 (95% CI 0.34–0.69)
Non-expert dermatologist (*n* = 14 studies)	Summary estimate AI	85.4% (95% CI 78.9–90.2%)	78.5% (95% CI 70.6–84.8%)	3.98 (95% CI 2.89–5.49)	0.18 (95% CI 0.13–0.27)
	Non-experts	76.4% (95% CI 71.1–80.9%)	67.1% (95% CI 57.2–75.6%)	2.32 (95% CI 1.71–3.14)	0.35 (95% CI 0.27-0.46)
Expert dermatologist (*n* = 16 studies)	Summary estimate AI	86.3% (95% CI 80.4–90.7%)	78.4% (95% CI 71.1–84.3%)	3.99 (95% CI 2.97-5.37)	0.17 (95% CI 0.12–0.25)
	Experts	84.2% (95% CI 76.2–89.8%)	74.4% (95% CI 65.3–81.8%)	3.29 (95% CI 2.31-4.67)	0.21 (95% CI 0.13–0.34)

Abbreviations: *LR* + = positive likelihood ratio; *LR*−= negative likelihood ratio.

Only 1 prospective study met the inclusion criteria and was included in the meta-analysis.

### AI vs overall clinicians’ meta-analysis

**Fig. 3:**
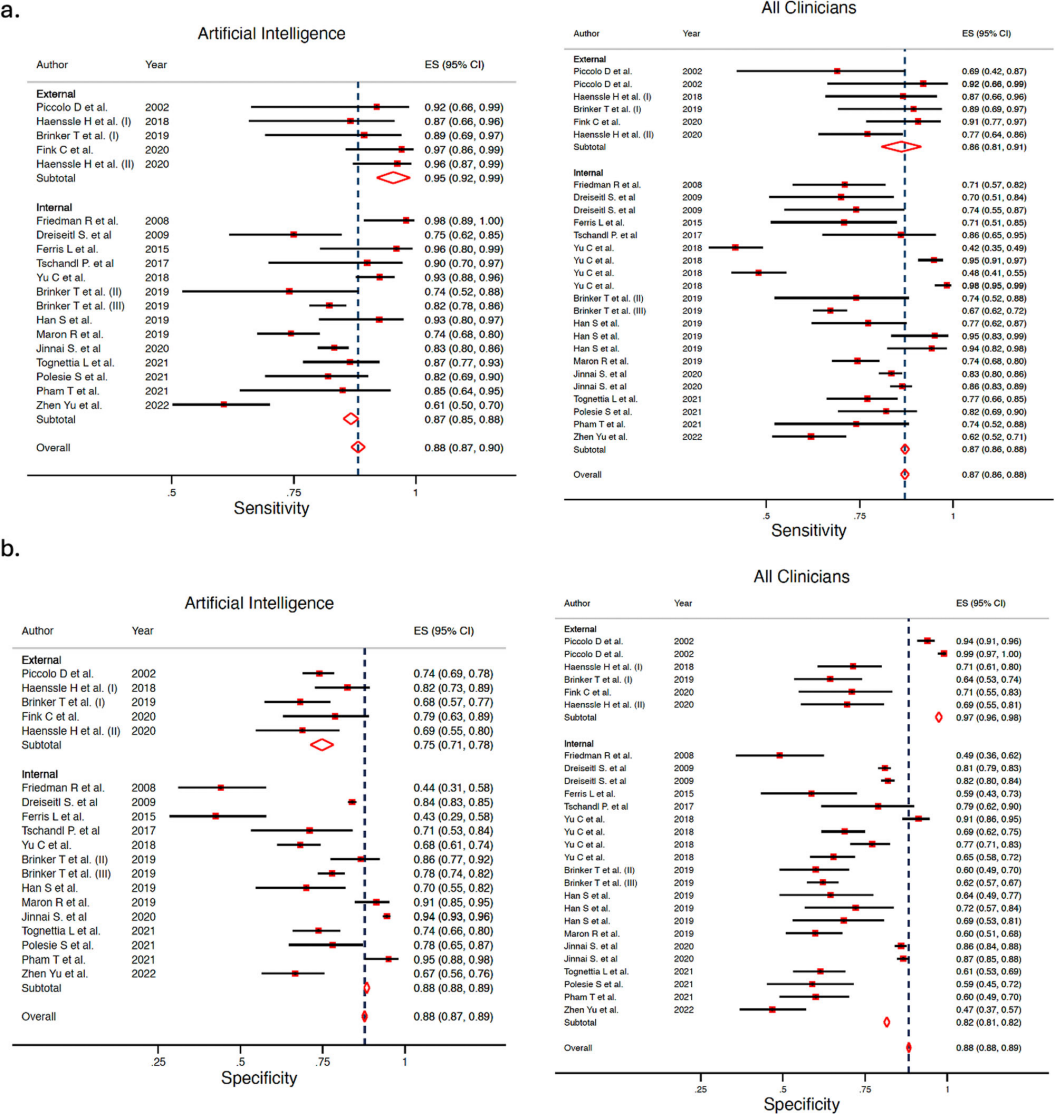
Forest plot detailing the sensitivity and specificity for all groups of clinicians (‘overall’) and artificial intelligence algorithms from each study included in the meta-analysis according to type of test set (external vs internal). **a** Sensitivity for artificial intelligence (left) and all clinicians (‘overall’) (right). **b** Specificity for artificial intelligence (left) and all clinicians (‘overall’) (right).

**Fig. 4:**
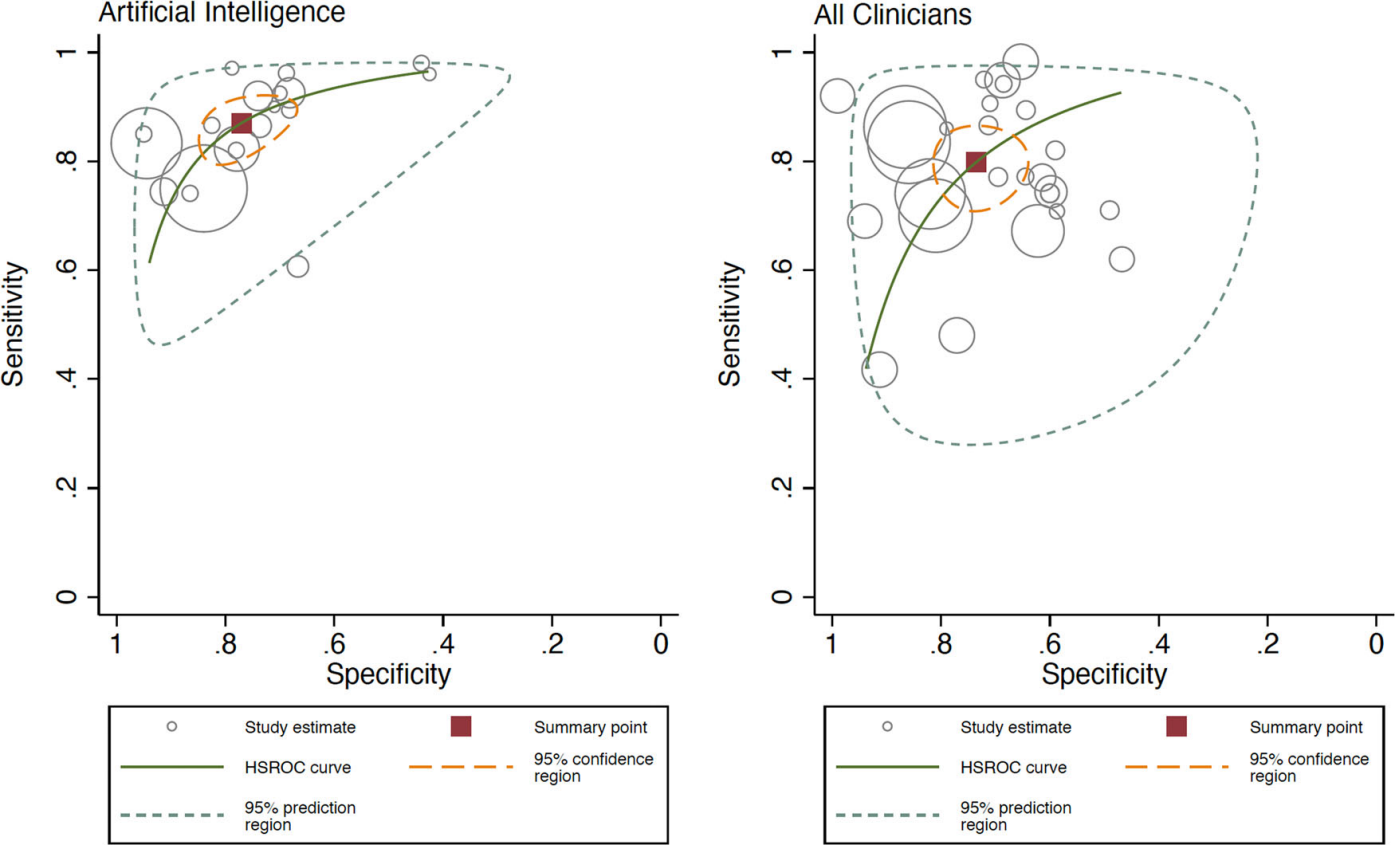
Hierarchical ROC curves of studies for comparing performance between artificial intelligence algorithms (left) and all group of clinicians (right). ROC receiver operating characteristic. Each circle size represents the individual study sample size (circle size is inversely related to study variance).

When analyzing the whole group of clinicians, not accounting for expertise level, AI obtained a Sn 87.0% (95% CI 81.7–90.9%) and Sp 77.1% (95% CI 69.8–83.0%), and overall clinicians obtained a Sn 79.8% (95% CI 73.2–85.1%) and Sp 73.6% (95% CI 66.5–79.6%), with a statistically significant difference for both Sn and Sp, according to the likelihood ratio test (*p* < 0.001 for both Sn and Sp). The Forest plot is available in Fig. 3a, b. The ROC curve shapes confirmed the prior differences (Fig. 4). Supplementary Fig. 1a, b shows the sub analysis adjusted for retrospective vs prospective design.

### AI vs generalists clinicians’ meta-analysis

**Fig. 5:**
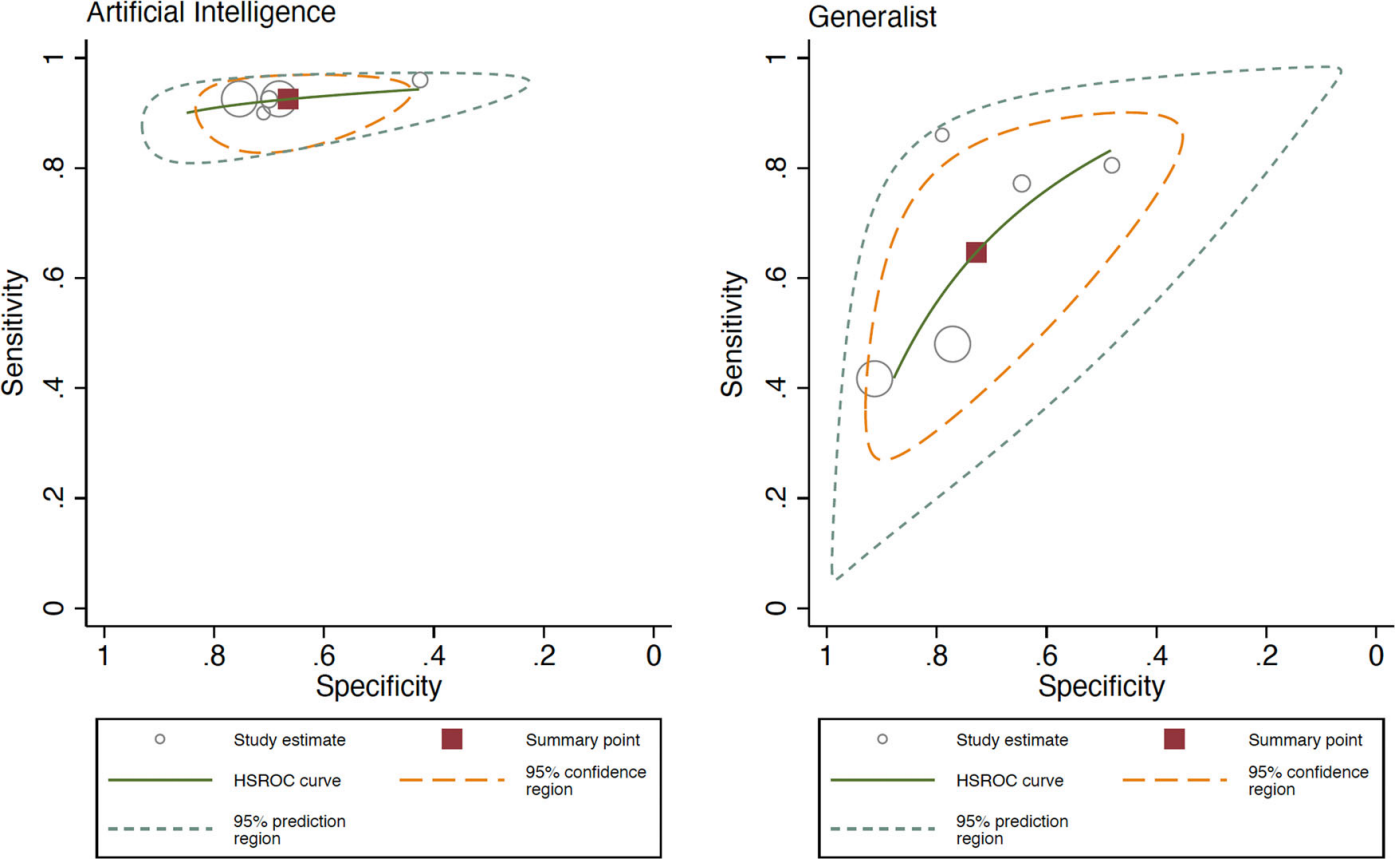
Hierarchical ROC curves of studies for comparing performance between artificial intelligence algorithms (left) and generalists (right). ROC receiver operating characteristic. Each circle size represents the individual study sample size (circle size is inversely related to study variance).

**Fig. 6:**
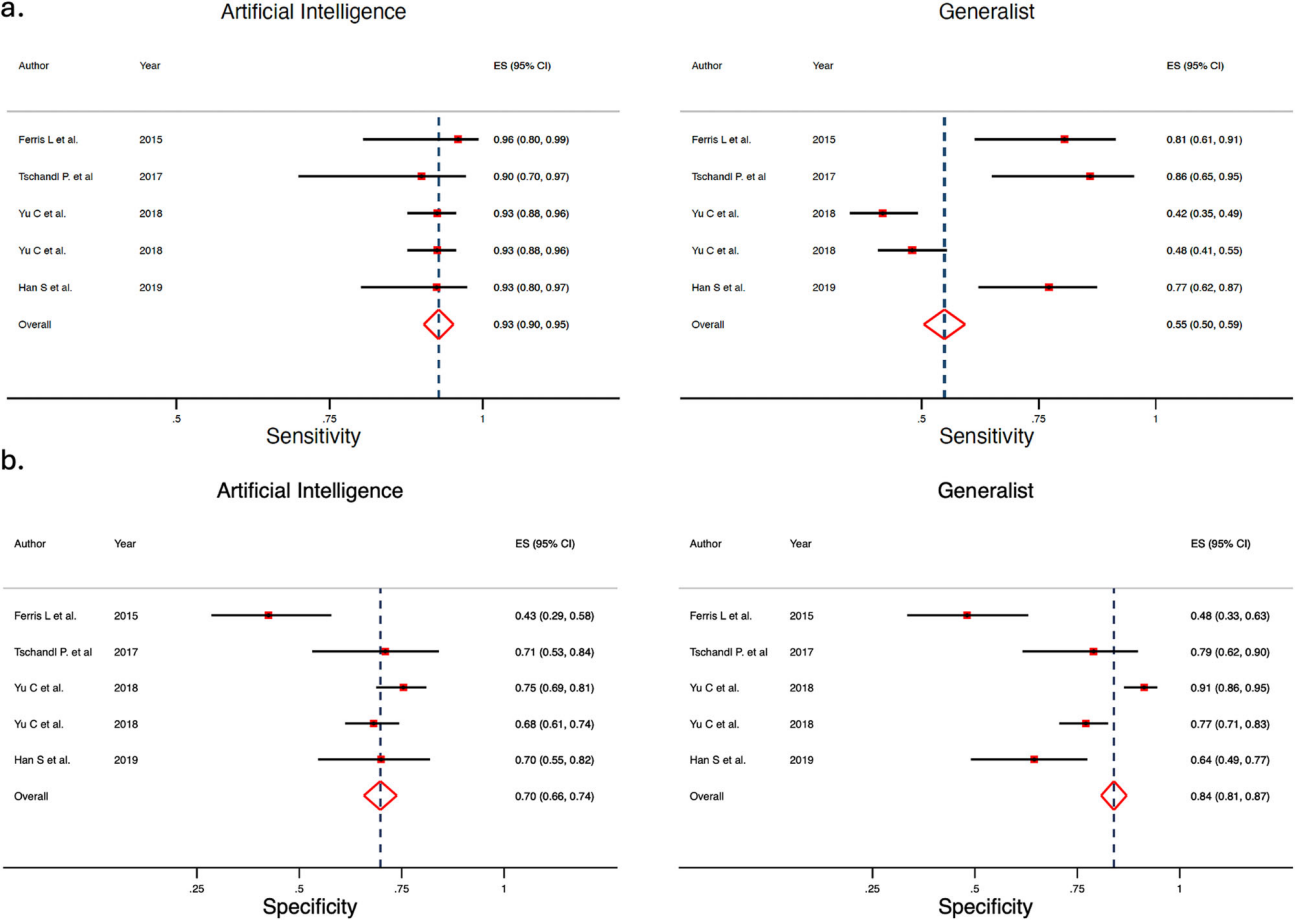
Forest plots of studies showing artificial intelligence vs generalists sensitivity and specificity. **a** Sensitivity for artificial intelligence (left) and for generalists (right). **b** Specificity for artificial intelligence (left) and for generalists (right).

When analyzing the AI performance vs generalists, AI obtained a Sn 92.5% (95% CI 88.9–94.9%) and Sp 66.5% (95% CI 56.7–75.0%), and generalists a Sn 64.6% (95% CI 47.1–78.9%) and Sp 72.8% (95% CI 56.7–84.5%), the difference being statistically significant for both Sn and Sp, according to the likelihood ratio tests (*p* < 0.001 for both). The ROC curve shapes confirmed the prior differences, with higher heterogeneity and wider confidence interval for generalists (Fig. 5). Subgroup analysis comparing internal vs external test set was not possible given all included studies were performed using internal test set in this subgroup (Fig. 6a, b).

### AI vs non-expert dermatologists’ meta-analysis

**Fig. 7:**
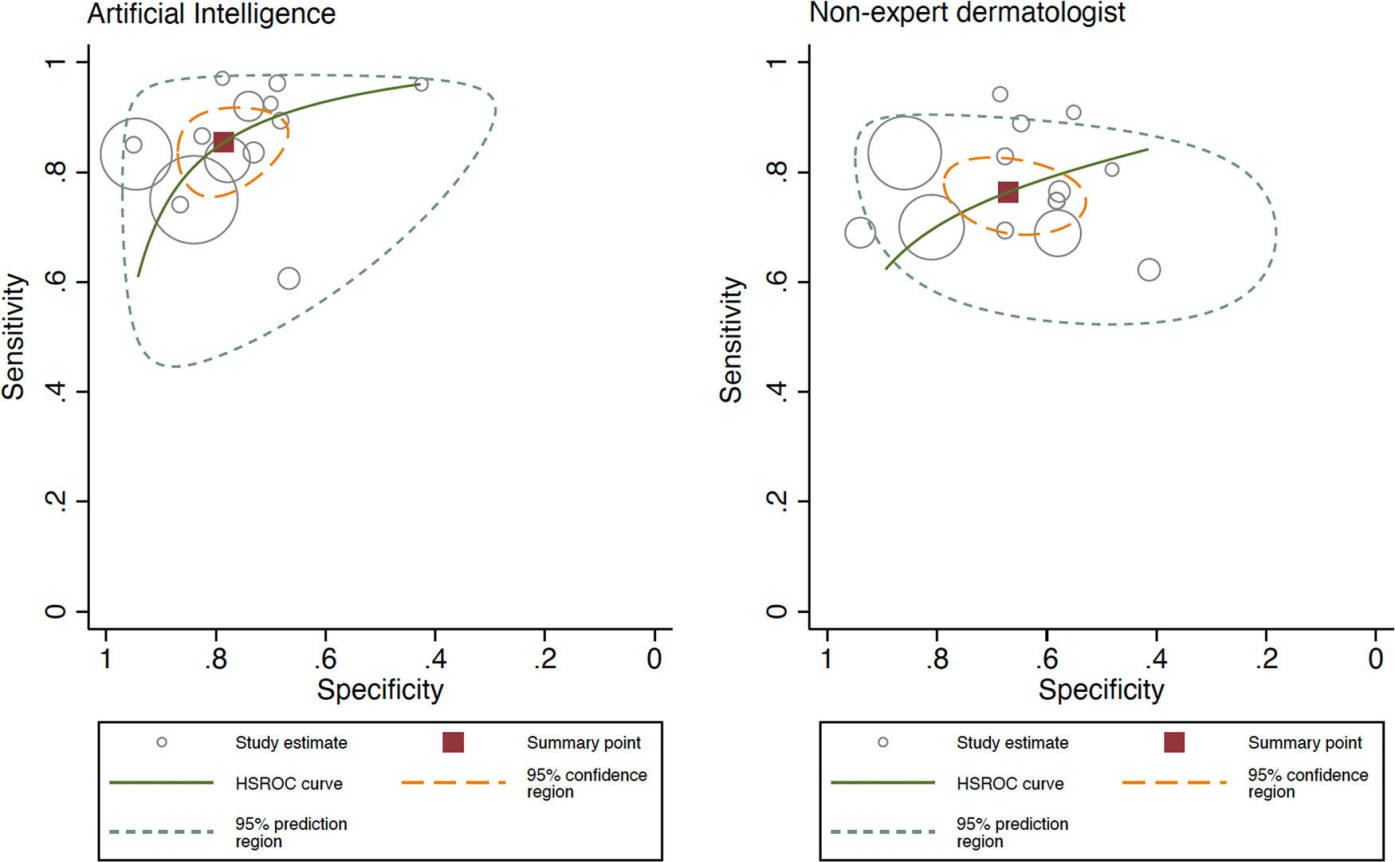
Hierarchical ROC curves of studies for comparing performance between artificial intelligence algorithms (left) and non-expert dermatologists (right). ROC receiver operating characteristic. Each circle size represents the individual study sample size (circle size is inversely related to study variance).

**Fig. 8:**
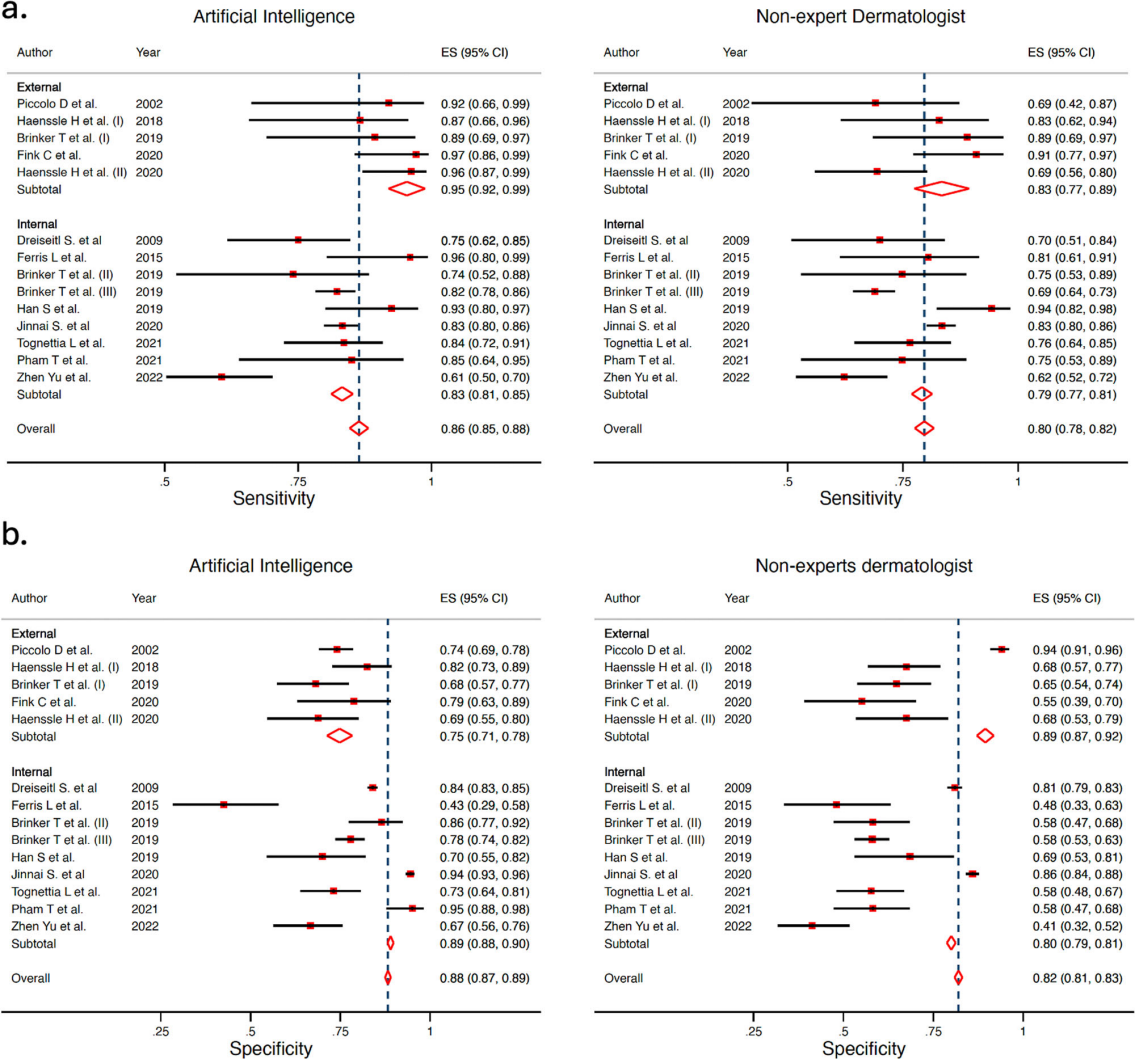
Forest plots of studies showing artificial intelligence vs non-expert dermatologists sensitivity and specificity according to type of test set (external vs internal). **a** Sensitivity for artificial intelligence (left) and for non-expert dermatologists (right). **b** Specificity for artificial intelligence (left) and for non-expert dermatologists (right).

AI obtained a Sn 85.4% (95% CI 78.9–90.2%) and Sp 78.5% (95% CI 70.6–84.8%), while non-expert dermatologists obtained Sn 76.4% (95% CI 71.1–80.9%) and Sp 67.1% (95% CI 57.2–75.6%), with a statistically significant difference, both in Sn and Sp (*p* < 0.001 for both). The ROC curve shapes confirmed these results (Fig. 7). The Forest plot is available in Fig. 8a, b. In the internal vs external test set subgroup analysis (Fig. 8a, b), AI achieved better Sn in the external test set, while greater Sp with an internal test set. For non-expert dermatologists, no changes in Sn were observed; however, they achieved better Sp in the external test set. In the prospective vs. retrospective subgroup analysis (Supplementary Fig. 2), only 1 prospective study met the inclusion criteria and was included in the meta-analysis. A trend towards better Sn in retrospective versus prospective studies was observed.

### AI vs expert dermatologists’ meta-analysis

**Fig. 9:**
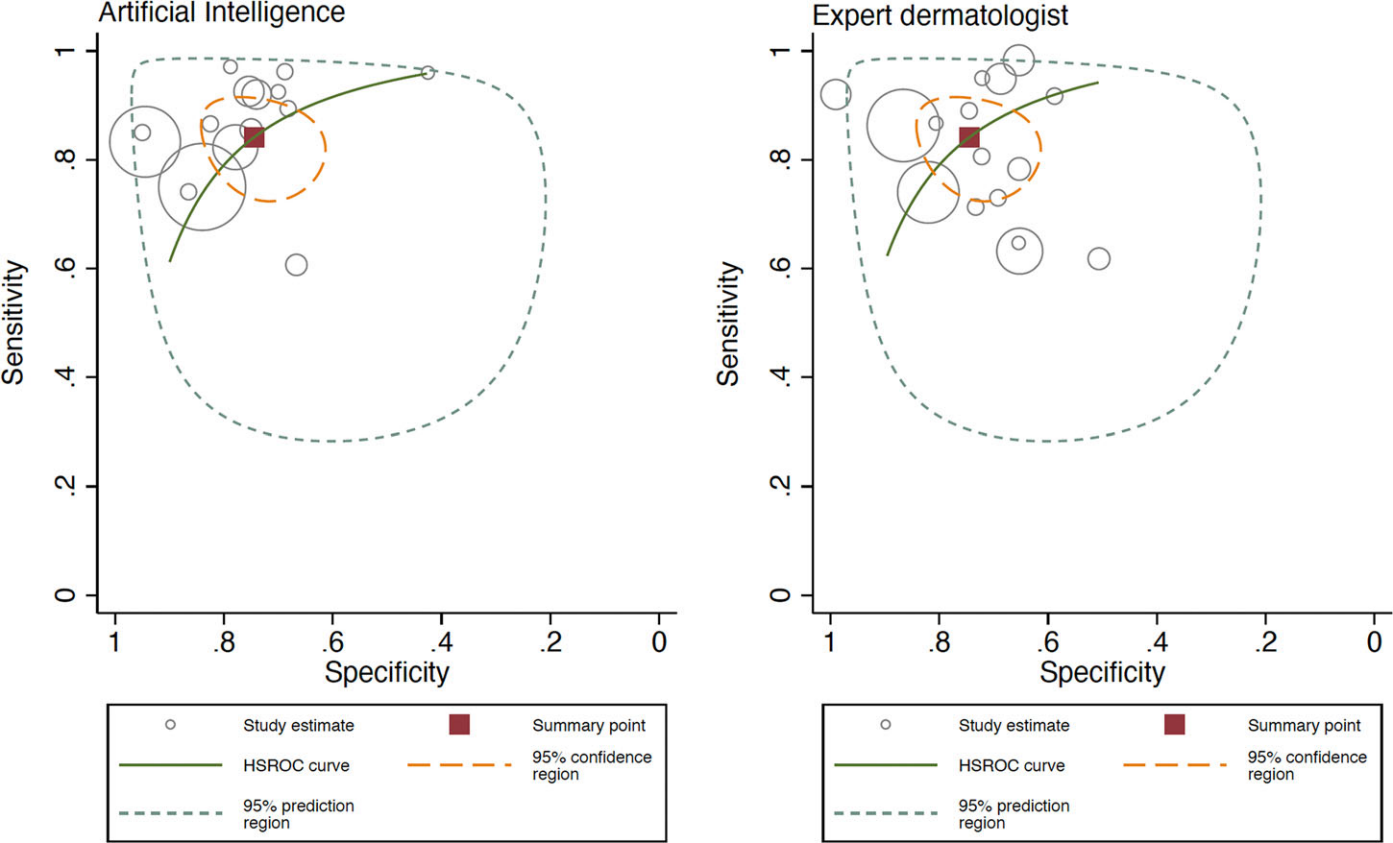
Hierarchical ROC curves of studies for comparing performance between artificial intelligence algorithms (left) and expert dermatologists (right). ROC receiver operating characteristic. Each circle size represents the individual study sample size (circle size is inversely related to study variance).

**Fig. 10:**
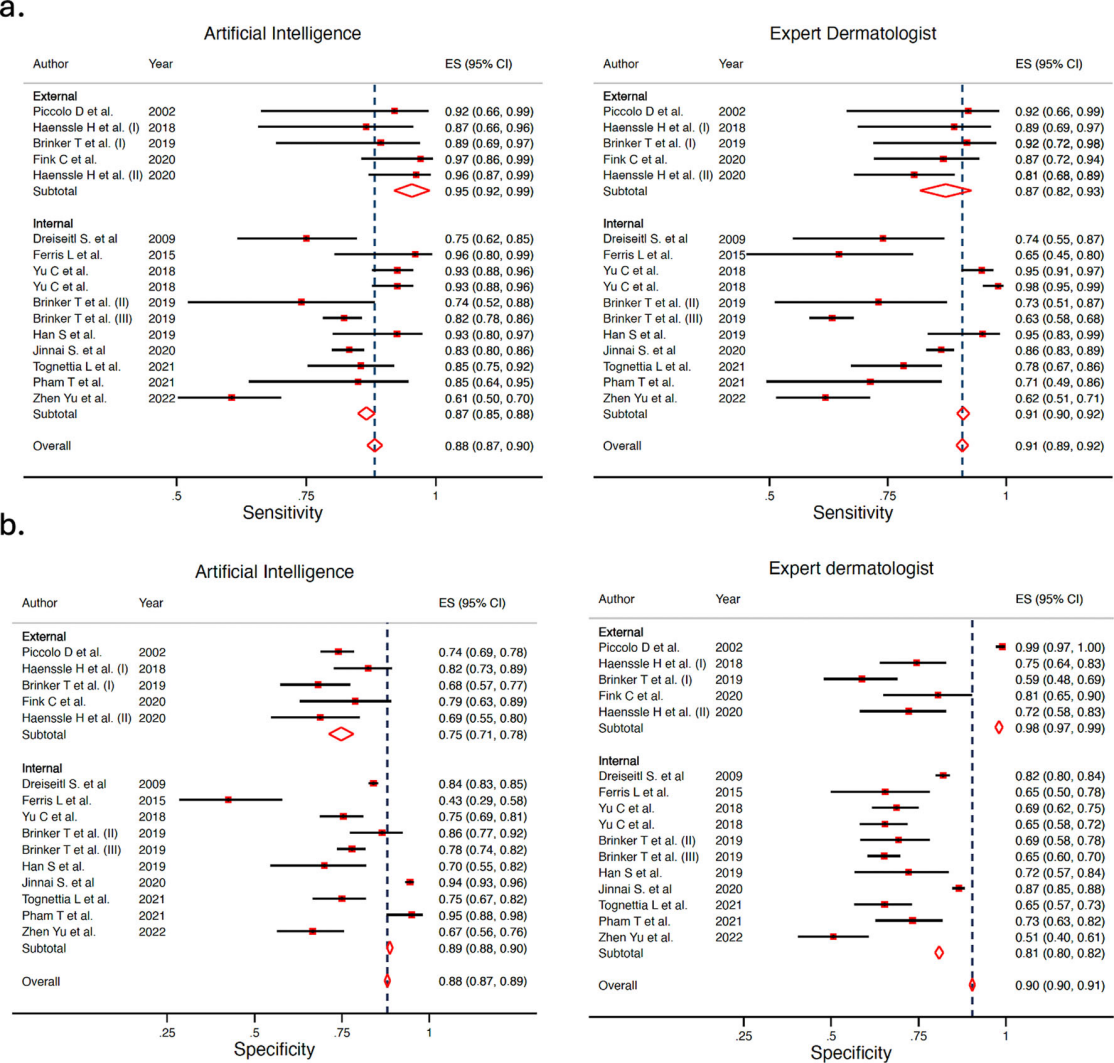
Forest plots of studies showing artificial intelligence vs expert dermatologists sensitivity and specificity according to type of test set (external vs internal). **a** Sensitivity for artificial intelligence (left) and expert dermatologists (right). **b** Sensitivity for artificial intelligence (left) and for expert dermatologists (right).

AI obtained a Sn 86.3% (95% CI 80.4–90.7%) and Sp 78.4% (95% CI 71.1–84.3%), and expert dermatologists a Sn 84.2% (95% CI 76.2–89.8%) and Sp 74.4% (95% CI 65.3–81.8%), this difference was statistically significant for both Sn and Sp, according to the likelihood ratio test (*p* < 0.001 for both). The ROC curve shapes were comparable for both AI and expert dermatologists, with narrow confidence intervals (Fig. 9). The subgroup analysis by internal vs external test set showed that AI had better Sn in external test set while Sp was better for internal test set. For expert dermatologists there was no difference in Sn; Sp was better in external test set (Fig. 10a, b). The subgroup analysis regarding study design, retrospective vs. prospective (Supplementary Fig. 3), found only one study.

## Discussion

In the present study, we found an overall Sn and Sp of 87% and 77% for AI algorithms and an overall Sn of 79% and Sp of 73% for all clinicians (‘overall clinicians’) when performing a meta-analysis of the included studies. Differences between AI and all clinicians were statistically significant. Performance between AI algorithms vs specialists was comparable between both groups. The difference between AI performance (Sn 92%, Sp 66%) and the generalists subgroup (Sn 64%, Sp 72%) was more marked when compared to the difference between AI and expert dermatologists. In studies that evaluated AI-assistance (‘augmented intelligence’), overall diagnostic performance of clinicians was found to improve significantly when using AI algorithms^62–64^. This improvement was more important for those clinicians with less experience. This is in line with this meta-analysis’ results where the difference was greater for generalist than for expert dermatologists and opens an opportunity for AI assistance in the group of less-experienced clinicians. To the best of our knowledge, this is the first systematic review and meta-analysis on the diagnostic accuracy of health-care professionals versus AI algorithms using dermoscopic or clinical images of cutaneous neoplasms. The inclusion of a meta-analysis is key to better understanding, quantitatively, the current state-of-the-art of AI algorithms for the automated diagnosis of skin cancer.

In general, the included studies presented diverse methodologies and significant heterogeneity regarding the type of images included, the different classifications, the characteristics of the participants, and the methodology for presenting the results. This is important to consider when analyzing and attempting to generalize and meta-analyze the obtained findings and should be taken into consideration when interpreting this study results. Research in AI and its potential applications in clinical practice have increased exponentially during the last few years in different areas of medicine, not only in dermatology^65^. Other systematic reviews have also reported that, in experimental settings, most algorithms are able to achieve at least comparable results when compared with clinicians; however, they also describe similar limitations as those described here^66–69^. Only a few studies have evaluated the role of AI algorithms in real clinical scenarios in dermatology. Our study confirms that only 5.7% of studies were prospective and only one of the prospective studies was suitable for meta-analysis^62,63^. This contrasts with recent data in other medical areas showing an increase in the clinical use of AI^70^ and highlights the relevance of understanding the role of AI in skin cancer and dermatology. However, prospective studies pose a real challenge for AI algorithms to become part of daily clinical practice as they face specific tests such as ‘out-of-distribution’ images or cases.

Based on this systematic review and meta-analysis results, several challenges have been evidenced when applying AI in clinical practice. First, databases are essential when training an AI algorithm. Small databases, inclusion of only specific populations, or limited variation in skin phototypes, limits the extrapolation of results^71–73^. The lack and underrepresentation of certain ethnic groups and skin types in current datasets has been mentioned as a potential source of perpetuation healthcare disparity^73^. Based on the results of our systematic review, we can confirm that most algorithms have been trained using the same datasets over and over in at least half of the studies. This translates into lack of representation of specific groups. The diversity of techniques and camera types (e.g. professional vs smartphones) used to capture images and their quality, possible artifacts such as pencil marks, rulers or other objects, are variables that must also be considered when evaluating the performance of AI algorithms^71,72,74^. A second limitation is the lack of inclusion of metadata in the AI algorithms. In the real world, we manage additional layers of information from patients, including demographic data, personal and family history, habits, evolution of the disease, and a complete physical examination, including palpation, side illumination, and not only 2-D visual examination. These elements are important to render a correct differential diagnosis and to guide clinical decision-making, and so far, very few AI models incorporate them. Therefore, real-world diagnosis is different from static 2-D image evaluations. Regarding the design of human evaluation in experimental and retrospective studies, in most cases it aims to determine whether a lesion is benign or malignant, or to provide a specific diagnosis. This differs from clinical practice in a real-life setting, in which decisions are generally behavioral, whether following up, taking a biopsy or removing a lesion, beyond exclusively providing a specific diagnosis based on the clinical evaluation. The scarce available prospective studies that account for this real-world clinical evaluation makes generalization of these positive results of AI mainly based on retrospective studies restricted. In addition, the management of patient information and privacy, and legal aspects of regulation regarding the application of AI-based software in clinical practice, also represents an emerging challenge^75^.

The current evidence gathered from this article supports collaboration between AI and clinicians (‘augmented intelligence’), especially for non-expert physicians. In the future, AI algorithms are likely to become a relevant tool to improve the evaluation of skin lesions by generalists in primary care centers, or clinicians with less access to specialists^63^. AI algorithms could also allow for prioritization of referral or triage, improving early diagnosis. Currently, there are undergoing studies evaluating the application of AI algorithms in real clinical settings, which will demonstrate the applicability of these results in clinical practice. The first prospective randomized controlled trial by Han et al.^62^, showed that when a group of clinicians used AI assistance, the diagnosis accuracy improved. This improvement was better for generalists. The results of this recent randomized clinical trial partially confirm the potentially positive role of AI in dermatology. These results also confirm that the benefit is more pronounced for generalists, aligning with the findings of the present meta-analysis.

With the aim of reducing the current barriers, we propose to generate and apply guidelines with standardization of the methodology for AI studies. One proposal is the Checklist for Evaluation of Image-Based Artificial Intelligence Reports in Dermatology, published by Daneshjou et al.^76^. These guidelines should include the complete workflow and start from the moment images are captured to protocols on databases, experience of participants, statistical data, definition on how to measure accuracy, among many others. This will allow us to compare different studies and generate better quality evidence. For example, Esteva et al.^52^. defined ‘overall accuracy’ as the average of individual inference class accuracies, which might differ from others. In addition, it is mandatory to collaborate with international collaborative databases (e.g. ISIC, available at www.isic-archive.com) to provide accessible public benchmarks and ensure repeatability and the inclusion of a diverse group of skin types and ethnicities to avoid for underrepresentation of certain groups. These strategies would make current datasets more diverse and generalizable.

The main strengths of the present study were the extensive and systematic search in 3 databases, encompassing studies from early AI days up to the most recently published studies, the strict criteria applied for the evaluation of studies and extraction of data, following the available guidelines for systematic reviews, and the performance of a meta-analysis, that allows for quantitatively assess the current AI data.

Limitations include the possibility of not having incorporated articles available in databases other than the ones included, or in other languages, thus constituting selection bias. Also, AI is a rapidly evolving field, and new relevant articles might have emerged while analyzing the data. To the best of our knowledge, no landmark studies were published in the meantime. Publication bias cannot be ruled out, since it is more likely that those articles with statistically significant results were to be published. Also, as shown in our results, more than half of the studies (64.1%) utilized the same public databases (e.g. ISIC and HAM10000), generating a possible overlap of the images in the training and testing group. Furthermore, most studies used the same dataset for training and testing the algorithm (73.6% used an internal test set) which might further bias the results. As observed in the subgroup analysis of the present study, there were differences in estimated Sn and Sp for both AI and clinicians depending on whether an internal vs. external test set was used. However, these were post-hoc analysis and should be interpreted with caution. External test set is key for proper evaluation of AI algorithms^6^ to ‘validate’ that the algorithm will retain its performance when presented with data from other datasets. Limited details regarding humans’ assessment by readers were available and could also affect the results. We also grouped all skin cancers as one group for analysis, variations in accuracy exists for different skin cancers (e.g. melanoma vs basal cell carcinoma vs squamous cell carcinoma) for humans and for AI algorithms. The application of QUADAS-2 shows a potential information bias, as it is an operator-dependent tool which generates subjectivity and qualitative results. Regarding the meta-analysis, we faced two main limitations. Firstly, the heterogeneity between studies makes it difficult to interpret or generalize the results obtained. Secondly, due to the lack of necessary data, the number of studies included in the meta-analysis was reduced when compared to the studies included in the systematic review. Finally, there was a minimal number of prospective studies included in the systematic review and only one was subjected to the meta-analysis and therefore, those results must be interpreted with caution. Nevertheless, in this post-hoc analysis prospective studies showed worse performance of AI algorithms compared to clinicians confirming the relevance of the complete physical examination and other clinical variables such as history, palpation, etc. This also shows a lack of real-world data published given most studies were retrospective reader studies.

## Conclusion

This systematic review and meta-analysis demonstrated that the diagnostic performance of AI algorithms was better than generalists, non-expert dermatologists, and despite being statistically significant, AI algorithms were comparable to expert dermatologists in the clinical practice as the differences were minimal. As most studies were performed in experimental settings, future studies should focus on prospective, real-world settings, and towards AI-assistance. Our study suggests that it is time to move forward to real-world studies and randomized clinical trials to accelerate progress for the benefit of our patients. The only randomized study available has shown a better diagnosis accuracy when using AI algorithms as ‘augmented intelligence’^62^. We envision a fruitful collaboration between AI and humans leveraging the strengths of both to enhance diagnostic capabilities and patient care.

### Supplementary information

Supplementary figures

STATA Codes

Dataset 1

## Data Availability

All metadata are available as supplementary material.

## Code availability

Codes are available as supplementary material.
