# Author Correction: A systematic review and meta-analysis of artificial intelligence versus clinicians for skin cancer diagnosis

**DOI:** 10.1038/s41746-024-01138-0

**Published:** 2024-05-24

**Authors:** Maria Paz Salinas, Javiera Sepúlveda, Leonel Hidalgo, Dominga Peirano, Macarena Morel, Pablo Uribe, Veronica Rotemberg, Juan Briones, Domingo Mery, Cristian Navarrete-Dechent

**Affiliations:** 1 Department of Dermatology, Escuela de Medicina, Pontificia Universidad Católica de Chile, Santiago, Chile; 2 Universidad Catolica-Evidence Center, Cochrane Chile Associated Center, Pontificia Universidad Católica de Chile, Santiago, Chile; 3 Melanoma and Skin Cancer Unit, Escuela de Medicina, Pontificia Universidad Católica de Chile, Santiago, Chile; 4 Dermatology Service, Department of Medicine, Memorial Sloan Kettering Cancer Center, New York, NY USA; 5 Department of Oncology, Escuela de Medicina, Pontificia Universidad Católica de Chile, Santiago, Chile; 6 Department of Computer Science, Pontificia Universidad Católica de Chile, Santiago, Chile

**Keywords:** Skin cancer, Translational research, Melanoma

Correction to: *npj Digital Medicine* 10.1038/s41746-024-01103-x, published online 14 May 2024

In the Acknowledgements section, the funding source ‘Partial funding was obtained from La Fondation La Roche Possay Research Awards.’ should have read ‘Partial funding was obtained from La Fondation La Roche Possay Research Awards. ANID - Millennium Science Initiative Program ICN2021_004”. The original article has been corrected.

