# Supplementary material for: A systematic review and meta-analysis of artificial intelligence versus clinicians for skin cancer diagnosis
Source: NPJ Digit Med. 2024 May 14;7:125. doi: 10.1038/s41746-024-01103-x (PMC11094047; doi:10.1038/s41746-024-01103-x)
Supplement: Supplementary file 1 — Supplementary figures [file 41746_2024_1103_MOESM1_ESM.pdf]

Supplementary files:

a.

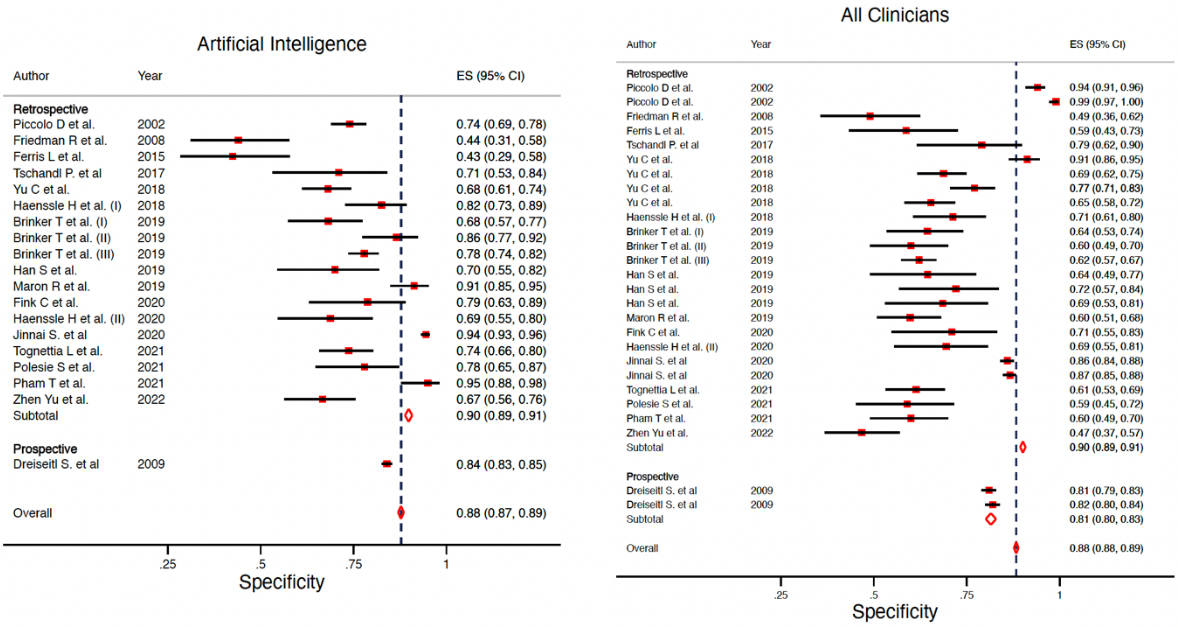

b.

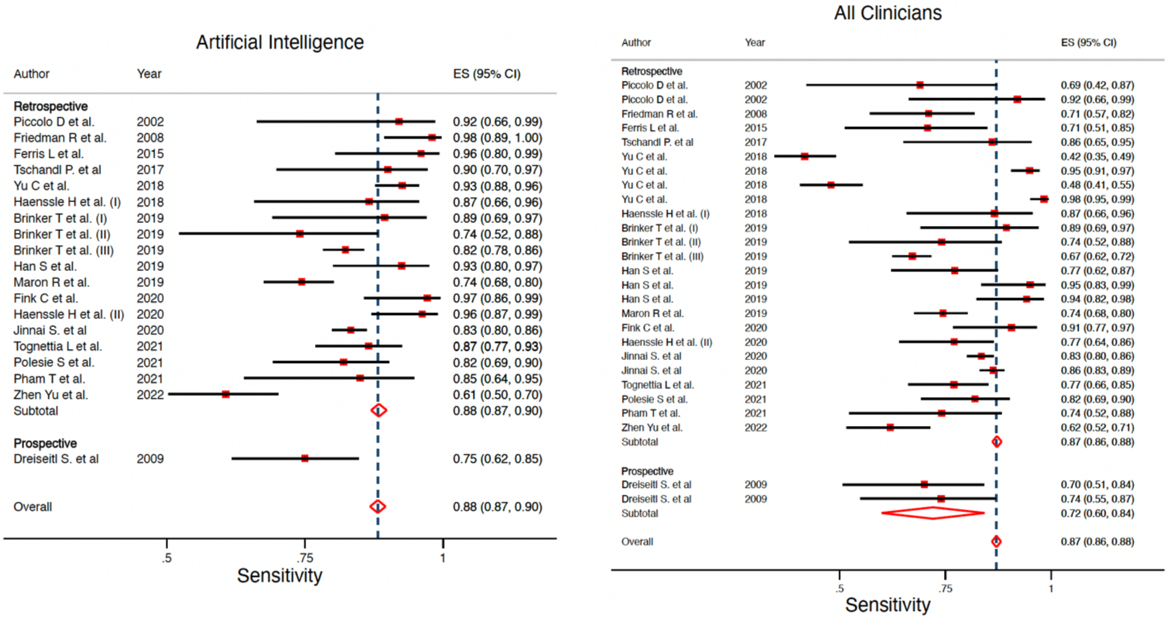

Supplementary Figure 1: Forest plot detailing the sensitivity and specificity for all groups of clinicians ('overall') and AI algorithms from each study stratified by study design ("retrospective vs prospective"). **a.** Sensitivity for artificial intelligence (left) and all clinicians ('overall') (right). **b.** Specificity for artificial intelligence (left) and all clinicians ('overall') (right).

a.

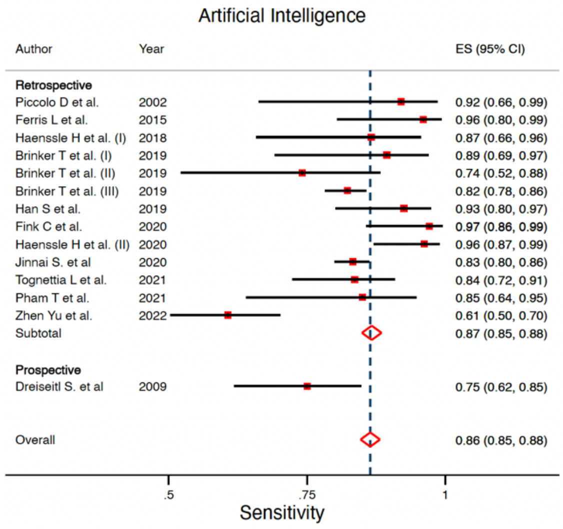

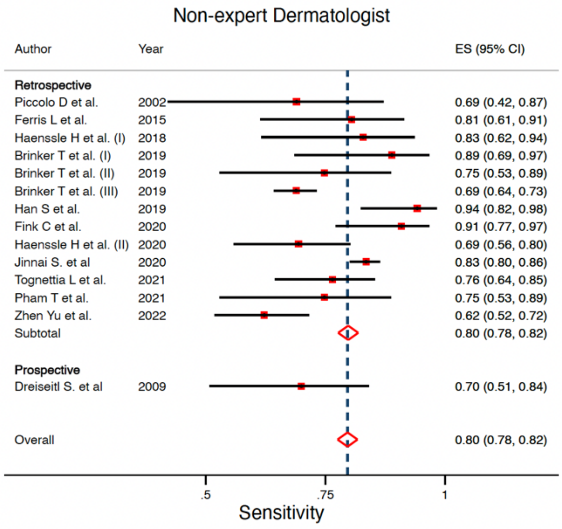

b.

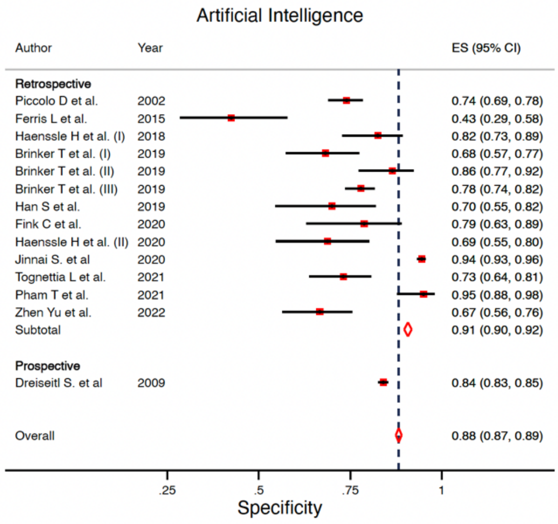

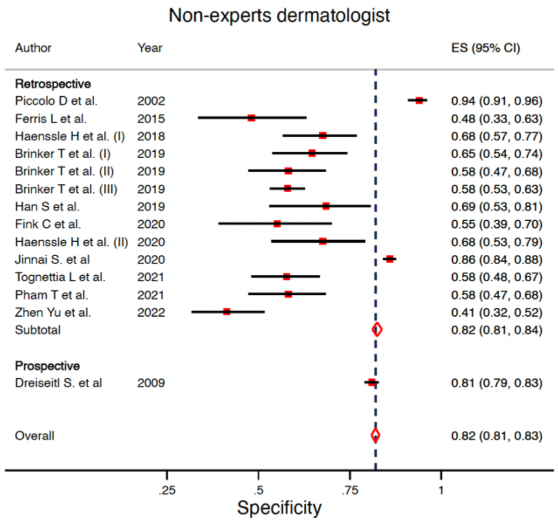

Supplementary Figure 2: Forest plots of studies showing artificial intelligence vs non-expert dermatologists sensitivity and specificity stratified by study design ("retrospective vs prospective"). **a.** Sensitivity for artificial intelligence (left) and for non-expert dermatologists (right). **b.** Specificity for artificial intelligence (left) and for non-expert dermatologists (right).

a.

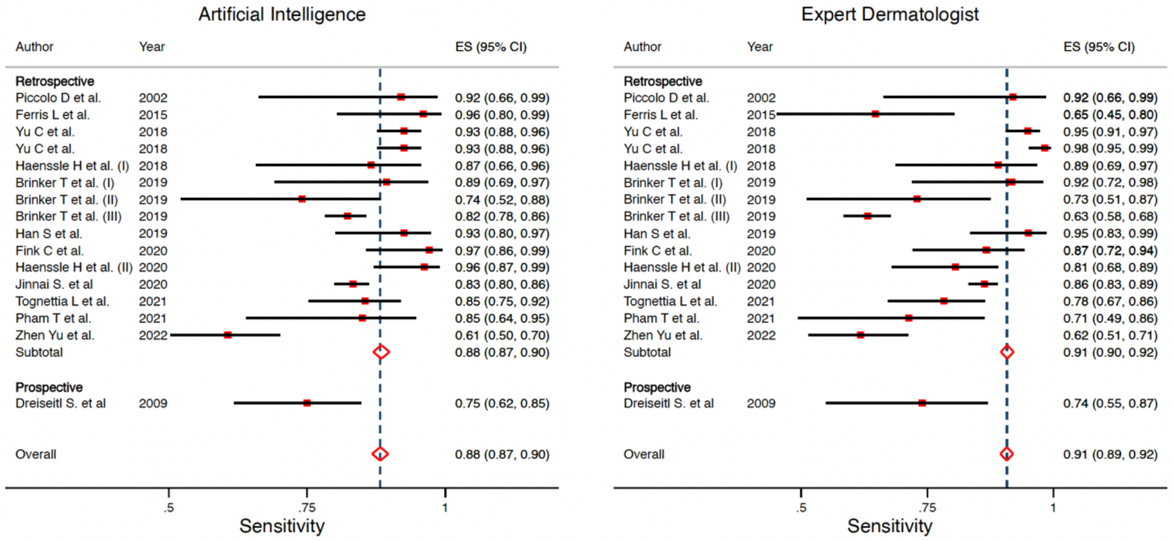

b.

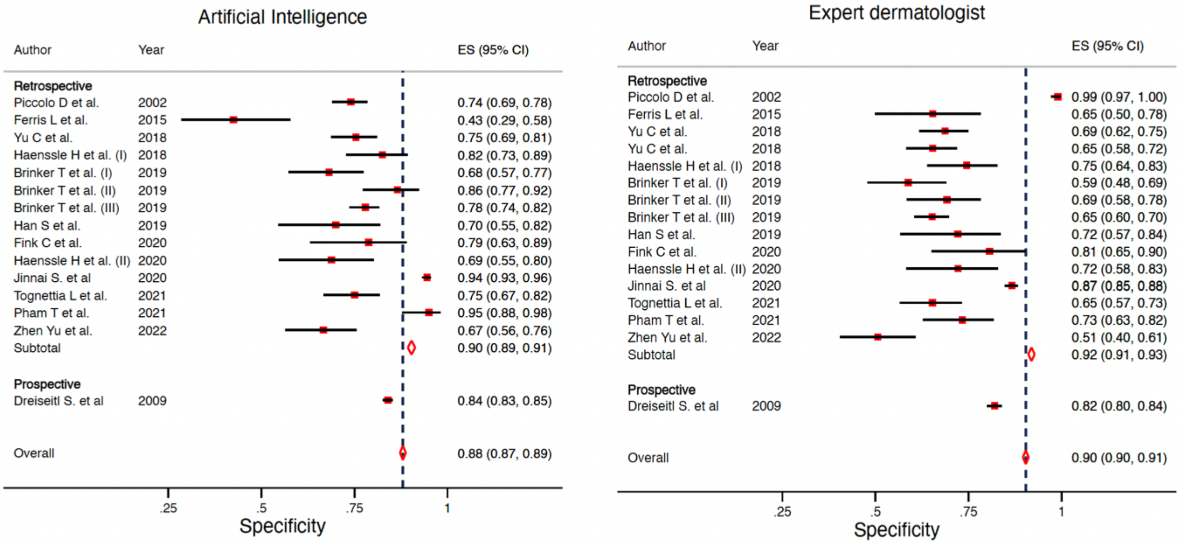

Supplementary Figure 3: Forest plots of studies showing artificial intelligence vs expert dermatologists sensitivity and specificity stratified by study design ("retrospective vs prospective"). **a.** Sensitivity for artificial intelligence (left) and expert dermatologists (right). **b.** Sensitivity for artificial intelligence (left) and for expert dermatologists (right).
