# Supplementary material for: A systematic review and meta-analysis of artificial intelligence versus clinicians for skin cancer diagnosis
Source: NPJ Digit Med. 2024 May 14;7:125. doi: 10.1038/s41746-024-01103-x (PMC11094047; doi:10.1038/s41746-024-01103-x)
Supplement: Supplementary file 2 — STATA Codes [file 41746_2024_1103_MOESM2_ESM.docx]

encode test, gen(methodtype)

label list methodtype

metandi TP FP FN TN if methodtype==1

metandiplot TP FP FN TN if methodtype==1, saving(test1 , replace)

metandi TP FP FN TN if methodtype==2

metandiplot TP FP FN TN if methodtype==2, saving(test2, replace)

gr combine test1.gph test2.gph

*********************************************************************************

gen Npos= TP + FN

gen Nneg= TN + FP

rename autor Author

rename año Year

rename imagenes Imaging

rename internalexternal Testset

rename tipoestudio Design

rename Expertiz Clinicians

label variable Author "Author"

label variable Year "Year"

label variable Imaging "Imaging"

label variable Testset "Testset"

label variable Design "Design"

label variable Clinicians "Clinicians"

metaprop TP Npos if methodtype==1, fixed label(namevar=Author) lcols(Author Year Testset) xlab(.5,.75,1)xline(0, lcolor(black)) subti("Artificial Intelligence", size(2)) xtitle ("Sensitivity",size(2)) olineopt(lcolor(red)lpattern(shortdash)) plotregion(icolor(white)) diamopt(lcolor(red)) pointopt(msymbol(x)msize(0))boxopt(msymbol(S) mcolor(red)) astext(50) texts(120) by(Testset) sortby(Year) dp(2) nowt nohet

metaprop TN Nneg if methodtype==1, fixed label(namevar=Author) lcols(Author Year Testset) xlab(.25,.5,.75,1)xline(0, lcolor(black)) subti("Artificial Intelligence", size(2)) xtitle ("Specificity",size(2)) olineopt(lcolor(red)lpattern(shortdash)) plotregion(icolor(white)) diamopt(lcolor(red)) pointopt(msymbol(x)msize(0))boxopt(msymbol(S) mcolor(red)) astext(50) texts(120) nohet by(Testset) sortby(Year) dp(2) nowt

********************************************************************************

metaprop TP Npos if methodtype==2, fixed label(namevar=Author) lcols(Author Year Testset) xlab(0.25,.5,.75,1)xline(0, lcolor(black)) subti("All", size(2)) xtitle ("Sensitivity",size(2)) olineopt(lcolor(red)lpattern(shortdash)) plotregion(icolor(white)) diamopt(lcolor(red)) pointopt(msymbol(x)msize(0))boxopt(msymbol(S) mcolor(red)) astext(50) texts(120) nohet by(Testset) sortby(Year) dp(2) nowt

metaprop TN Nneg if methodtype==2, fixed label(namevar=Author) lcols(Author Year Testset) xlab(0.25,.5,.75,1)xline(0, lcolor(black)) subti("All", size(2)) xtitle ("Specificity",size(2)) olineopt(lcolor(red)lpattern(shortdash)) plotregion(icolor(white)) diamopt(lcolor(red)) pointopt(msymbol(x)msize(0))boxopt(msymbol(S) mcolor(red)) astext(50) texts(120) nohet by(Testset) sortby(Year) dp(2) nowt

********************************************************************************

gen long n1= TP + FN

gen long n0=FP+TN

gen long true1=TP

gen long true0=TN

gen long recordid= _n

reshape long n true, i(recordid) j(sens)

gen byte spec=1-sens

sort Author test

gen setest1=0

gen sptest1=0

gen setest2=0

gen sptest2=0

replace setest1=1 if test=="test1" & sens==1

replace sptest1=1 if test=="test1" & spec==1

replace setest2=1 if test=="test2" & sens==1

replace sptest2=1 if test=="test2" & spec==1

meqrlogit true sens spec if test=="test1", nocons ||Author: sens spec, nocons cov(un) binomial(n) refineopts(iterate(3)) intpoints(5) variance

meqrlogit true sens spec if test=="test2", nocons ||Author: sens spec, nocons cov(un) binomial(n) refineopts(iterate(3)) intpoints(5) variance

meqrlogit true sens spec, nocons ||Author: sens spec, nocons cov(un) binomial(n) refineopts(iterate(3)) intpoints(5) variance nolr

estimates store A

meqrlogit true setest1 setest2 sptest1 sptest2, nocons ||Author: sens spec, nocons cov(un) binomial(n) refineopts(iterate(3)) intpoints(5) variance nolr

estimates store B

lrtest A B

meqrlogit true sens sptest1 sptest2, nocons ||Author: sens spec, nocons cov(un) binomial(n) refineopts(iterate(3)) intpoints(5) variance nolr

estimates store C

lrtest B C

meqrlogit true setest1 setest2 spec, nocons ||Author: sens spec, nocons cov(un) binomial(n) refineopts(iterate(3)) intpoints(5) variance nolr

estimates store D

lrtest B D

meqrlogit true setest1 setest2 sptest1 sptest2, nocons ||Author: setest1 sptest1, nocons cov(un) ||Author: setest2 sptest2, nocons cov(un) binomial(n) refineopts(iterate(3)) intpoints(5) variance nolr

estimates store E

lrtest B E

lrtest A E

*********************************************************************************

matrix list e(V)

nlcom diff_sensitivity: invlogit(_b[setest1])-invlogit(_b[setest2])

nlcom diff_specificity: invlogit(_b[sptest1])-invlogit(_b[sptest2])

nlcom log_relative_sensitivity: log(invlogit(_b[setest1]))-log(invlogit(_b[setest2]))

nlcom log_relative_specificity: log(invlogit(_b[sptest1]))-log(invlogit(_b[sptest2]))

capture program drop renamematrix

program define renamematrix, eclass

matrix mb = e(b)

matrix mv = e(V)

matrix colnames mb = logitsetest1:_cons logitsetest2:_cons logitsptest1:_cons logitsptest2:_cons

matrix colnames mv = logitsetest1:_cons logitsetest2:_cons logitsptest1:_cons logitsptest2:_cons

matrix rownames mv = logitsetest1:_cons logitsetest2:_cons logitsptest1:_cons logitsptest2:_cons

ereturn post mb mv

end

renamematrix

_diparm logitsetest1, label(Sensitivity test1) invlogit

_diparm logitsetest2, label(Sensitivity test2) invlogit

_diparm logitsptest1, label(Specificity test1) invlogit

_diparm logitsptest2, label(Specificity test2) invlogit

_diparm logitsetest1 logitsptest1, label(LR+ test1) ci(log) function(invlogit(@1)/(1-invlogit(@2))) derivative(exp(@2-@1)*invlogit(@1)^2/invlogit(@2) exp(@2)*invlogit(@1))

_diparm logitsetest2 logitsptest2, label(LR+ test2) ci(log) function(invlogit(@1)/(1-invlogit(@2))) derivative(exp(@2-@1)*invlogit(@1)^2/invlogit(@2) exp(@2)*invlogit(@1))

_diparm logitsetest1 logitsptest1, label(LR- test1) ci(log) function((1-invlogit(@1))/invlogit(@2)) derivative(exp(-@1)*invlogit(@1)^2/invlogit(@2) exp(-@1-@2)*invlogit(@1))

_diparm logitsetest2 logitsptest2, label(LR- test2) ci(log) function((1-invlogit(@1))/invlogit(@2)) derivative(exp(-@1)*invlogit(@1)^2/invlogit(@2) exp(-@1-@2)*invlogit(@1))
